# Raman Sensing and Its Multimodal Combination with Optoacoustics and OCT for Applications in the Life Sciences

**DOI:** 10.3390/s19102387

**Published:** 2019-05-24

**Authors:** Merve Wollweber, Bernhard Roth

**Affiliations:** 1 Laser Zentrum Hannover e.V., Industrial and Biomedical Optics Department, Hollerithallee 8, 30419 Hannover, Germany; 2 Hannover Centre for Optical Technologies, Leibniz University Hannover, Nienburger Str. 17, 30167 Hannover, Germany; bernhard.roth@hot.uni-hannover.de; 3 Cluster of Excellence PhoenixD, Leibniz University Hannover, Welfengarten 1, 30167 Hannover, Germany

**Keywords:** Raman spectroscopy, optical coherence tomography, optoacoustics, dermoscopy, multimodal sensing and imaging

## Abstract

Currently, many optical modalities are being investigated, applied, and further developed for non-invasive analysis and sensing in the life sciences. To befit the complexity of the study objects and questions in this field, the combination of two or more modalities is attempted. We review our work on multimodal sensing concepts for applications ranging from non-invasive quantification of biomolecules in the living organism to supporting medical diagnosis showing the combined capabilities of Raman spectroscopy, optical coherence tomography, and optoacoustics.

## 1. Optical Spectroscopy & Applications in the Life Sciences—A Perfect Match

The objects of research in the life sciences are usually characterized by a complex structure and composition as well as considerable individual variance. Generally, it is challenging—and often even impossible so far—to achieve extensive, reliable, and in-depth analysis of biological materials non-invasively. Optical methods are well suited for biomedical and environmental research as they usually allow non-invasive and fast measurements that may even be used for in situ studies, i.e., to investigate where and how a research object naturally occurs, without the need for sampling or sample preparation.

Consequently, recent decades have seen increasing interest in optical approaches for biological applications. Investigation of liquids such as water or blood, cell or tissue cultures, or even whole plants, animals, or humans is possible. Optical tomography and microscopy are already widely applied in the life sciences, with laser scanning fluorescence microscopy being the flagship imaging modality today. Other optical modalities have been spreading, especially in biomedical applications. Applied methods include two-photon microscopy [1], optical coherence tomography (OCT) [2], and super-resolution microscopy (also called nanoscopy) via structured illumination [3], stimulated emission depletion (STED) or single molecule localization microscopy (SMLM) [4,5,6,7] to name some of the most popular. Furthermore, also hybrid techniques such as optoacoustic imaging, also called photoacoustic imaging, have been developed towards clinical application [8].

Fluorescence labeling provides molecular contrast and information in some of the imaging modalities already mentioned. Optical spectroscopy based on autofluorescence, absorption or Raman scattering [9] may be applied to obtain chemical or molecular information from a target tissue by intrinsic contrast alone, i.e., without the need for fluorescent labels.

Naturally, there is no single approach that can answer all research questions and generally, strong efforts must be made to adapt and develop a modality for a new application. Strengths and weaknesses of current imaging and spectroscopic approaches must be critically reviewed and chosen for each given task [10]. The review articles by Teodori et al. [10], Kim et al. [11] and Das et al. [12] may be consulted for a more detailed treatise on the advantages and weaknesses of the single methods. Besides, research objectives in the life sciences are often inherently multi-scale. In answer to this, there has been a trend towards combination of different modalities in recent years aimed at obtaining complementary information. For example, optoacoustic imaging has been combined with ultrasound imaging, fluorescence imaging, OCT, and multi-photon microscopy [11]. Similar efforts have also been made for Raman spectroscopy combining it for example with fluorescence or elastic scattering imaging, OCT, phase imaging, and mass spectrometry [12]. Qiao et al. [13] recently proposed a dual-mode combination of OCT and photoacoustic imaging for optical biopsies of skin while Rao et al. [14] promote the perspective of combined OCT and Raman spectroscopy to support cancer diagnosis. Despite this long list of combinations that have already been proposed or tested, so far, these efforts and achievements have still been highly selective, proposing or presenting customized solutions only for single applications. Especially in vivo application often still poses a major challenge. Therefore, except for fluorescence imaging, method development is often at the stage of proof of concept in a physics laboratory rather than becoming a standard tool for biological or medical research.

In line with the current trends of optical method development for life science research reported so far, this review article focuses on our own work on advancing and developing spectroscopic and imaging modalities to investigate specific research objectives within this field.

Some of these objectives demand to question comprehensively how an otherwise well-established method needs to be handled in the context of a new application.

Section 2 presents own work on making Raman spectroscopy fit the demands of investigations on tissue, unicellular organisms, or specific molecules under physiologic conditions. In this context, we developed several experimental and analytical tools for Raman spectroscopy of biological samples (Section 2.1). Here, we studied the effects of fixation procedures (Section 2.1.1) and of the molecular environment in in vitro samples on their Raman spectroscopic characteristics with the aim of finding conditions for fixed or artificial samples to exhibit near to identical Raman characteristics as the respective in vivo target. Such assessment is crucial for referencing and method validation. To reliably extract information from Raman spectroscopic data and allow a maximal degree of comparability also for data from different experimental setups, we developed data processing tools covering tasks from customized background elimination to correction of the spectra for device response and spectral calibration (Section 2.1.2). In addition, we critically evaluated different multivariate approaches for data analysis (Section 2.1.3). Building on this preparatory work, we analyzed Raman spectra of two types of molecules that are of general interest in biological research: proteins (Section 2.2) and carotenoids (Section 2.3). With respect to proteins, the extracted information at the molecular level enabled us to study protein function, i.e., connexin gating, and the structural changes connected with it (Section 2.2.1) and to establish an approach for bacteria identification in the native biofilm (Section 2.2.2). The Raman spectrum of carotenoids heavily depends on the molecular environment, so interpretation of Raman spectra from carotenoid mixtures—as usually found in the living organism—needs careful interpretation (Section 2.3.1). If this is taken into account, however, Raman studies can, for example, reveal carotenoid transitions and composition in algae via principal component analysis of resonantly enhanced spectra or via analysis of the Raman resonance profiles (Section 2.3.2).

Another focus of our work presented in this review is to transfer modalities, which are typically used for imaging or spectral characterization and categorization, into non-invasive in vivo measurement tools. This approach was followed for Raman spectroscopy, optoacoustics, and OCT. In principle, Raman spectroscopy may not only be used to identify molecules or organisms but also to quantify molecule concentration. Attempts to do so regularly fail in connection with biological samples because of indeterminable spectral signal attenuation. To solve this, we worked on the development of non-invasive methods to measure absorption and scattering properties in vivo. Section 3 recapitulates our efforts and achievements concerning the reconstruction of depth-dependent absorption properties from optoacoustic data, covering forward (Section 3.1) and inverse (Section 3.2) solutions and pushing the approach towards application of optoacoustics for determination of the thickness of pigmented nevi in human skin (Section 3.3).

Finally, Section 4 summarizes our research activities concerning combination of different imaging or spectroscopic modalities. Here, our efforts are pursuing mainly two directions. On the one hand, we worked on combining different imaging modalities to merge their information content into one multimodal image representation. On the other hand, we use data such as the optical properties that had not been available from in vivo measurements before to support and improve the quality of information that can be deduced by another method. For either of these directions of research, it is crucial to have defined samples or tissue phantoms for method validation that meet the needs of all modalities involved, e.g., optical and acoustic specifications (Section 4.1). Such samples are highly useful to test the calculation of optical properties from optoacoustic data, see Section 3, or in developing strategies to recover pure, molecule-specific Raman spectra from measurements on complex samples where they are obscured by spectral attenuation (Section 4.2). The combined knowledge of in vivo optical properties together with simplified and well-designed in vitro samples may also be applied to the in-depth analysis of photobiological processes by aid of numerical models. As an example, we show work on the calculation of vitamin D${}_{3}$ production in human skin (Section 4.3). Such calculations on photoconversion of molecules may in the future be used to predict also the dynamic change of optical properties connected with the molecular changes—not only with regard to vitamin D but also, for example, with regard to, for example, the carotenoid transitions of the xanthophyll cycle as investigated in (Section 2.3.2). Section 4.4 deals with application of OCT and closes the circle of complementary and mutually supportive methods presented in this review. In Section 4.4.1, optical coherence tomography is explored as a measurement tool proposing a new numerical approach for extracting scattering properties from the OCT data in order to complement the information on absorption properties obtained by optoacoustics. This marks the next step towards absolute quantification of molecule concentration as it allows to also include scattering data in the numerical simulations of Raman attenuation and thus to significantly improve the model. OCT can not only be used to derive scattering coefficients but also to measure depth of diagnostically relevant features in human skin (Section 4.4.2) and this information may be combined with information from other modalities, i.e., Raman spectroscopy and optoacoustic depth profiling, to develop a new diagnostic tool for the assessment of pigmented nevi (Section 4.4.3).

Having presented these various aspects of our work on multimodal approaches towards in vivo investigations in the life sciences, we close with some concluding remarks on their future development.

## 2. Raman Spectroscopy

Raman spectroscopy probes the molecular vibrations of a given sample by detection of inelastically scattered photons. As vibrational and rotational energy levels are highly specific for a certain molecule, Raman spectra are often referred to as molecular fingerprints. They can be used to analyze biological samples even at a functional level making use of intrinsic contrast alone and without the need for labeling [15,16,17,18,19]. If—owing to the composition of the sample or to technical tricks—only a single type of molecule is addressed, (resonance) Raman spectroscopy allows detailed analysis of the electronic, vibrational, and even rotational energy states of the molecule as well as its conformation [20,21,22,23]. Few-component spectra may be analyzed to the single-component level and multi-component Raman spectra may still be taken as representative for the sample entity. Besides, the fact that non-invasive measurements are possible and that Raman spectroscopy per se requires no a priori knowledge about the sample boosted its use for label-free analysis of technical or biological samples.

Raman scattering cross-sections are orders of magnitude lower than the cross-sections for other photon-molecule processes such as absorption, elastic scattering or fluorescence making Raman signals comparably weak (see e.g., [24], Table 1.8). Furthermore, laser fluence, peak power and exposure need to be carefully controlled to avoid modification of the sample caused by the measurement or even sample degeneration or destruction. Therefore, generally, suitable enhancement methods are imperative for Raman spectroscopy in life science applications to overcome the challenges of low concentrations in in situ spectroscopy of biological samples. Various enhancement techniques are available and need to be chosen according to the specific application and—in practice—also based on available instrumentation. In coherent anti-stokes Raman spectroscopy (CARS) [25] and stimulated Raman spectroscopy (SRS) [26], coherent excitation is used to enhance weak Raman signals by a four-wave mixing process. Surface-enhanced (resonance) Raman spectroscopy takes advantage of plasmon resonances achieving strong local enhancement but necessitating introduction of nanoparticles into the sample [27,28]. Resonance Raman spectroscopy benefits from the strong increase of scattering cross-sections and ultimately of the Raman signal if the excitation wavelength is tuned close to an electronic transition of the target molecule [17]. In general, the Raman spectrum of a particular molecule is independent of the excitation wavelength. However, if the energy of the scattered photon matches the energy of an electronic transition of the molecule, absorption and scattering cross-sections are strongly increased and so is the intensity of specific lines in the respective Raman spectrum. This resonance effect may enhance the Raman signal by several orders of magnitude. Besides, target molecules may be deliberately selected and enhanced above others based on their resonance behavior. This can be used to specifically address target molecules and enhance their signal above a possibly complex background signal. The effect of increased absorption needs to be considered in resonance Raman spectroscopy. However, in biological samples, it is rather the matrix absorption that causes unwanted sample damage as absorbed energy in a single class of target molecules generally dissipates well in the samples that often have a high water content.

This section mainly focuses on research exploiting resonance enhancement for Raman analysis of biological samples. Besides showing examples of the information that can be gathered non-invasively via Raman spectroscopy from a given biological sample, the section also aims at presenting and discussing technical and analytical challenges connected with this approach and strategies to address them in order to obtain valid and reliable data.

### 2.1. Experimental and Analytical Tools for Raman Spectroscopy of Biological Samples

Even though the Raman spectrum of a molecule is an intrinsic and characteristic feature, it is important to always be aware that Raman measurement conditions can have a significant effect on the measured Raman signal especially when studying biological samples. The Raman signal of such samples may be affected by sample preparation such as fixation (Section 2.1.1). A minimum requirement for the comparability of spectra measured at different excitation wavelengths (as in resonance Raman spectroscopy) is the correction of Raman signals by the device response function to account for the spectral sensitivity of the detection setup (Section 2.1.2). As biological samples often exhibit strong fluorescence, customized background correction is highly useful to unmask the Raman signal in the collected spectrum (Section 2.1.2) even though it cannot eliminate fluorescence noise which can severely impair the quality of measured Raman spectra. After careful processing of the raw spectral data, multivariate analysis may be applied to extract or deduce the wanted information. However, this step also requires a careful choice of the best or at least a suitable approach with respect to the individual set of data (Section 2.1.3)

#### 2.1.1. Sample Preparation

As pointed out already, Raman spectroscopy is particularly attractive for life science applications as it does not require sample preparation such as labeling or fixation but is capable of in vivo and in situ measurement on native samples. Still, measurement of fixed or labeled samples may sometimes be desired. Fixed samples are needed to retain certain conditions, e.g., to stop metabolism of bacterial samples if a certain growth status or other condition at a specific point in time is to be documented. Moreover, fixation is often required for follow-up analysis with other methods such as fluorescent in situ hybridization (FISH) which is the standard method to identify bacteria.

There are several fixation methods and protocols available, so we evaluated some of the most prominent ones for their compatibility with subsequent Raman measurements [29]. Our work from 2011 is one of the very few studies investigating the effect of fixation on the Raman signal of single cells [30,31,32,33]. All these previous works studied human cell lines. Some more studies focused on effects in whole tissue.

**Figure 1:**
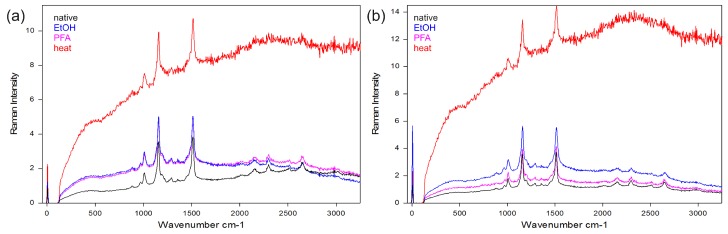
Resonant Raman spectra at excitation with 532 nm of the bacterial strains *rhodopseudomonas palustris* (**a**) and *rhodospirillum rubrum* (**b**) and the effect of treatment with different fixatives compared to the Raman spectrum of the native bacteria. The main Raman-active chromophore for both strains is the carotenoid spirilloxanthin with dominant lines at 1509 cm${}^{-1}$, 1151 cm${}^{-1}$, and 1004 cm${}^{-1}$ (reproduced with permission from [29]).

Based on measurements on three bacteria strains containing heme or carotenoid(s) as chromophores for identification, we found that even gentle heat fixation at 70 °C is not compatible with Raman spectroscopy as it causes a strongly increased background and a complete loss of the weaker Raman lines. As shown in Figure 1, only the three dominant carotenoid lines remain clearly visible.

On the other hand, cultures treated with formaldehyde (PFA) or ethanol (EtOH) for fixation display only small differences to the native bacteria in their Raman spectrum. Still, care must be taken not to exceed treatment times. For both fixatives, the signal-to-noise ratio decreases with treatment time. This effect is stronger for ethanol fixation where an ever-increasing fluorescent background is observed while the degenerative effect of PFA fixation on the obtained Raman signals saturates after some hours [29]. We also found that mounting bacterial samples on poly-L-lysine coated microscope slides, which are quite popular in the life science community as cell cultures adhere well to them, can decrease Raman signals. This effect was only prominent in cultures of bacteria with average cell sizes smaller than the axial diameter of the focus of the confocal microscope used in this study indicating that additional flattening of the target cells and the resulting decrease of measurement volume within the focus may be the reason for this effect.

The good preservation of Raman spectral features under formalin fixation agrees with the results presented in [30,31,32,33] for human cell cultures. Our work is the first to study fixation effects on the Raman fingerprints of bacteria and the first to study ethanol and heat fixation or the effect of coated coverslips in this respect. Only in 2017, Hobro et al. attempted at a more comprehensive review and study of fixation effects on Raman spectra of cells [34].

#### 2.1.2. Data Processing—Spectral Calibration, Absolute Intensities, and Background Correction

**Figure 2:**
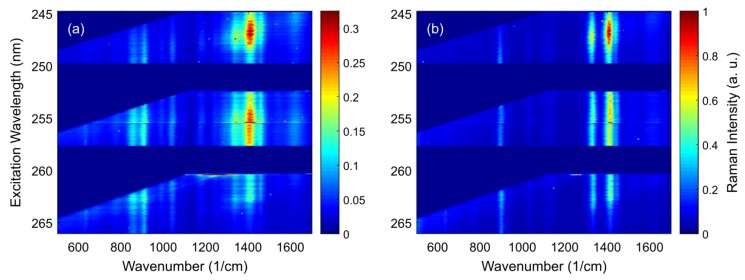
Excitation-emission maps (EEM) of the amino acids (**a**) proline and (**b**) glycine. Raman intensity is color-coded. Raman peaks appear as perpendicular lines. Dark blue regions in the map mark spectral ranges where no Raman signal could be measured due to lack of appropriate filters (reproduced with permission from [35]).

There are two levels of making use of the resonance effect in Raman spectroscopy. On a basic level—and this is by far the most frequently referred to when finding the term resonance Raman in the literature—experimenters capitalize on the resonance effect to enhance the signal of a specific molecule at a single excitation wavelength. However, at a more sophisticated and laborious level, it may also refer to investigating the resonance behavior of a target molecule. This second level requires excitation of Raman spectra at several wavelengths. Multiline lasers such as the Ar${}^{+}$-ion laser with multiple available lines in the blue to green wavelength range or laser systems that are spectrally tunable over a wide spectral range such as dye lasers and optical parametric oscillators (OPOs) provide suitable excitation sources. Detection is significantly more elaborate than in the case of single-wavelength enhancement. First, notch or long pass filters are necessary at every excitation wavelength to block the Rayleigh scattered photons, which would otherwise obscure the Raman signal causing unwanted stray light in the spectrograph. Alternatively, a triple-monochromator may be used providing maximal versatility at the cost of very low photon collection efficiency. Commercial filters are generally only available for standard laser wavelengths. They can be angle tuned by some nanometers, but this is by far not enough to achieve sufficient spectral coverage for truly continuous measurement of complete resonance Raman spectra throughout the visible and ultraviolet spectral range. Filters can be used at excitation wavelengths blue-shifted with respect to the nominal filter wavelength at the cost of low wavenumber Raman signals to minimize gaps in the coverage of the resonance range for a specific Raman-active molecule. In general, it is desirable to cover at least the Raman fingerprint region (500–1700 cm${}^{-1}$) with the transmission range of the filter. Figure 2 shows the resonance behavior of two amino acids in the form of excitation-emission maps.

Amino acids do not have large conjugated pi-electron systems, so their resonance conditions are usually met in the ultraviolet C range. As can be seen, full fingerprint spectra can only be recorded at very narrow excitation ranges a few nm wide. Therefore, in this study, even spectra covering only half of the fingerprint region were included to achieve a minimal level of continuity for the pre-resonance profiles of the higher wavenumber lines around 1400 cm${}^{-1}$ at least.

For high-resolution resonance Raman spectroscopy, spectral calibration poses a challenge depending on the applied excitation wavelengths and the specification of the spectrograph. At 250 nm in the ultraviolet C range, the Raman fingerprint region (500–1700 cm${}^{-1}$) only extends from 253–261 nm explaining the immediate cropping of the spectra when moving outside the specified laser wavelength for a given edge filter as shown by the dark blue areas in Figure 2. When measuring resonance spectra in the visible spectral range, the fingerprint region approximately spans 20 to 80 nanometers for deep blue to dark red excitation, respectively. At excitation with 500 nm Raman fingerprints are found between 513–546 nm. A high-resolution spectrograph, e.g., Andor Shamrock 500i, with 500 mm focal length, 1200 L/mm grating, and 27.6 mm wide CCD chip can image approximately 40 nm spectral range. Therefore, with blue or green excitation, a full fingerprint spectrum can be imaged at a time. However, when measuring resonance profiles, the excitation wavelength is tuned over 60–100 nm in the visible range and consequently the imaged spectral range needs to be adjusted every few nanometers. This is easily accomplished by means of a turnable grating turret. However, in our experience, the repeatability of the turret position and consequently of the spectral calibration at each turret angle is not sufficiently accurate. Therefore, spectral recalibration is necessary after every movement of the grating turret. We implemented two procedures for spectral recalibration. Ideally, spectral recalibration is accomplished by (co-)measurement of an external or internal standard. We followed this standard approach making use of the atomic emission lines of a neon lamp in [36]. Alternatively, the tunable excitation source can be used for spectral calibration when excitation is provided by the optical parametric oscillator in our experiments [35]. Here, the accuracy of the wavelength selection of the laser system limits the accuracy of the spectral calibration of the spectrograph. If no such standard is available and a set of Raman peak positions of the target molecule can be assumed to be fixed, a basic spectral adjustment may also be based on the Raman spectrum itself knowing the excitation wavelength. However, line positions preserve only relative information in such self-referencing datasets. It must be pointed out that such spectral recalibration is a challenge quite specific to our unique broadband excitation scheme in connection with high-resolution Raman spectroscopy.

A standard step in the post-processing of Raman spectral data is their normalization to make the spectra of the same target comparable even at differing experimental conditions. In contrast to all other methods of Raman spectroscopy, resonance Raman spectra must not be normalized independently but instead, great care must be taken to reliably measure absolute Raman intensities at comparable conditions. Otherwise, the signal property containing the resonance information will be lost. Therefore, only Raman measurements obtained at the same detection efficiency usually realized by maintaining a certain detection geometry and sensitivity may be combined to yield Raman resonance profiles and data needs to be normalized to the (accumulated) excitation energy. For continuous wave laser systems with stable output power, a basic stage of energy normalization can already be realized by keeping constant the integration time for a set of measurements. However, exact monitoring of accumulated laser power is advantageous making the obtained data better adaptable and comparable to other experimental conditions. For pulsed laser systems such as the OPO, close energy monitoring is prerequisite to obtain comparable spectra as most of these systems make use of multiple steps of frequency conversion. In the case of the VIS-OPO often used in the reviewed work, three consecutive non-linear stages lead to considerable variation in pulse-to-pulse energy which is of course further increased when using the second harmonic of the OPO signal output to excite Raman spectra in the ultraviolet range (as in Figure 2).

Beyond the mere collection efficiency of a Raman spectrometric probe, it is its spectral sensitivity of the setup that needs also be accounted for. Main factors are the reflectivity of gratings and mirrors and the quantum efficiency of the camera used for detection. We developed a procedure to acquire this spectral device response function measuring the intensity, i.e., the peak height, of a single laser pulse of the excitation laser as recorded by the spectrometer and relating it to its pulse energy measured via a power meter. A full spectrum of this relation yields a device response function which can be applied to correct the spectral Raman intensity as measured by the spectrograph [35].

To evaluate the presented preparatory work as well as the experimental and instrumental efforts for measuring resonance Raman excitation profiles, it should be pointed out that so far, there have been only a few attempts to go beyond the limits of multiple or multiline laser systems for investigation of resonance excitation profiles by other groups, such as Lewis et al. probing the excitation profile of bacteriorhodopsin by aid of a dye laser [37] or more recently Londero et al. presenting and discussing application of an OPO (10 ns pulse duration, 100 Hz repetition rate) in a Raman microscope [38]. The most extensive exploit of resonance Raman profiling was probably conducted by the groups of Hiroyoshi Nagae and Yasushi Koyama starting with a publication investigating the 2A${}_{g}^{-}$ energy of crystalline all-trans-spheroidene by analyzing its resonance Raman profile at low temperatures [39]. Even though determination of the excitation profile of the Raman resonance is rewarding, researchers still balk experimental and instrumental challenges and efforts such as lack of suitable laser systems and appropriate filters or the comprehensive evaluation of the detection system to allow reasonable determination of Raman intensities and reliable spectral calibration at different excitation wavelengths.

Fluorescence noise is a main factor obscuring Raman signals. Conventional ways to avoid fluorescence is excitation in the near-infrared region where electronic absorption and emission transitions are absent or in the ultraviolet range sufficiently below the onset of fluorescence emission which also benefits from much increased scattering cross-sections as compared to infrared excitation. In other spectral ranges, background correction is essential to extract Raman signals from the raw spectra, which are usually compromised by fluorescent background signals. In the case of resonance Raman spectroscopy, not only the background fluorescence from the multi-component constituents of the tissue, cell, or plant plays a role but also the fluorescence from the target molecule itself is substantial as resonance conditions for Raman scattering are in the majority of cases associated with near to optimal conditions for autofluorescence.

As background removal generally is a major issue for Raman spectroscopy, several approaches are in use owing to the diversity of experimental conditions. Some of them require special instrumentation. A method called shifted excitation spectral differences measures spectra at two close excitation wavelengths and capitalizes on the fact that fluorescence is broadband and independent of the exact excitation wavelength while Raman lines shift according to the excitation wavelength [40,41]. Time-gated approaches make use of the different lifetimes for scattering and fluorescence and collect only photons arriving early—before the onset of fluorescence emission—at the detector [42]. Our own experience applying the time-gated approach using an intensified charge-coupled device (ICCD) as the gated detector was that fluorescence lifetimes in biological samples are often too short to allow clear temporal separation of Raman scattering and fluorescence signals. These cases are examples where it may be beneficial to combine temporal separation with shifted excitation spectral differences as in [43]. Both approaches are demanding in terms of instrumentation. This is one reason post-processing of Raman spectra for background removal is highly popular but so far no universal approach is available. Polynomial curve fitting, first derivative methods, or Fourier-based algorithms are common, but tend to fail for data with irregularly shaped baselines, have difficulties in detecting signal peaks in low signal-to-noise spectra, or need well-tuned filter parameters, respectively. Schulze et al. [44] give an overview of classic baseline correction algorithms and their limitations.

**Figure 3:**
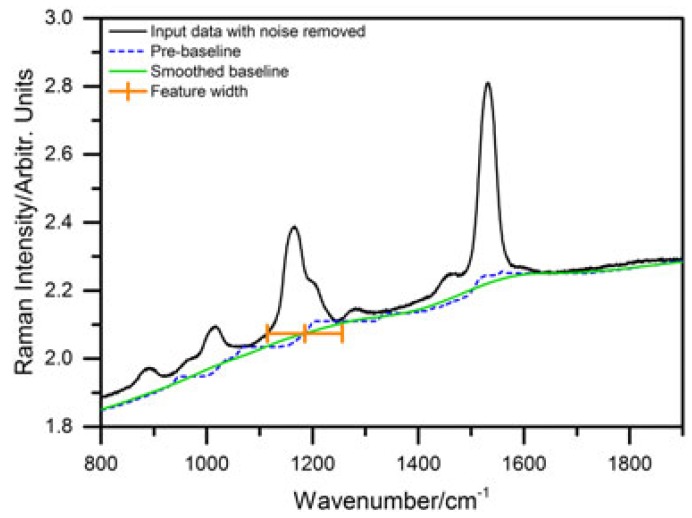
Smoothed measured Raman spectrum with corresponding feature width and the resulting pre-baseline. Smoothing the pre-baseline gives a (first) approximation of the true baseline. The smoothed baseline is subtracted from the unsmoothed measured spectrum completing the first iteration step (reproduced with permission from [45]).

To cope with the requirements of in vivo resonance Raman spectroscopy as in our applications, we developed a fast, and very efficient baseline correction algorithm for its particular needs [45]. We use an iterative approach in which every step involves a morphological operation together with mollification. Spectral intensity is smoothed by convolution with a mollifier kernel and a pre-baseline is formed by the footpoints of the smoothed data within a certain spectral width. This procedure can be imagined as fitting a horizontal line from the bottom into the experimental spectrum. The contact point of spectrum and horizontal line yields one point in the pre-baseline and the horizontal line is moved over the whole measured spectrum to create the complete pre-baseline from these contact points. Figure 3 illustrates the procedure.

The width of the horizontal line is determined by the width of significant features in the spectrum. It needs to be wider than the baseline width of the widest coalescent Raman lines. Iteration is stopped at the (first) elbow point of the relative area change between two successive iteration steps relative to the initial spectral area. This number of iterations leads to maximal background reduction at minimal occurrence of artifacts which may arise due to steep initial background slopes at high iteration numbers.

**Figure 4:**
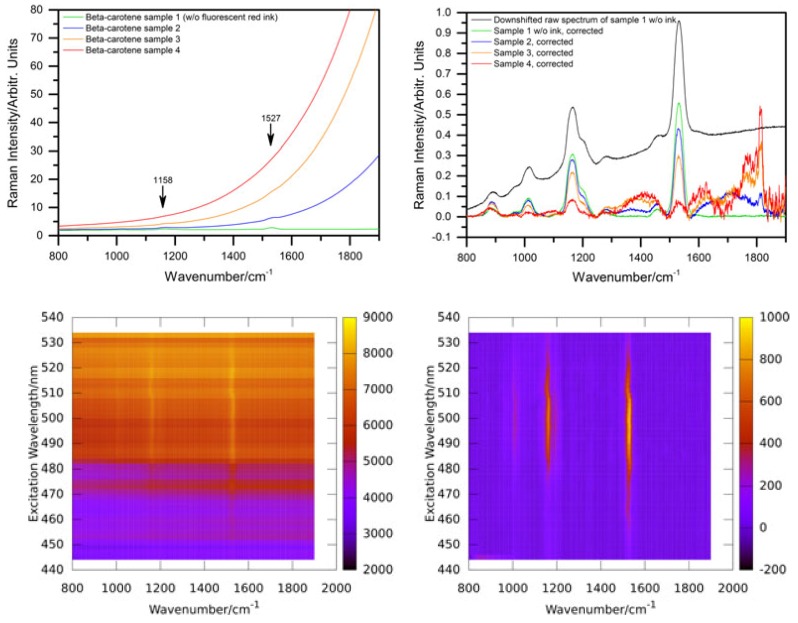
The **top** row shows raw (**left**) and processed (**right**) carotenoid Raman spectra in strongly fluorescent ink. The line position of two main carotenoid lines are marked by arrows in the original data set (**left** for orientation. The result of batch processing of a larger Raman data set is shown in the **bottom** row by excitation-emission maps of raw (**left**) and processed (**right**) Raman spectra (reproduced with permission from [45]).

The top row of Figure 4 shows results for spectra with very strong fluorescence. The algorithm is well suited for batch processing of large sets of irregularly shaped in vivo Raman spectra. The bottom row of Figure 4 shows excitation-emission maps of in vivo Raman measurements of an algae culture with a strong contribution from fluorescence taken under similar experimental conditions.

Our approach is well suited for baselines which cannot be handled by shape model-based traditional algorithms and where fully automated, solely morphological algorithms fail as evaluated comparing our new background removal algorithm with several other approaches [45]. One important aspect of our approach is that spectral features smaller than a chosen width are preserved under all circumstances to allow quantitative comparisons as in resonance Raman spectroscopy, which is important to evaluate coalescent Raman lines even with very low signal-to-noise ratio.

**Figure 5:**
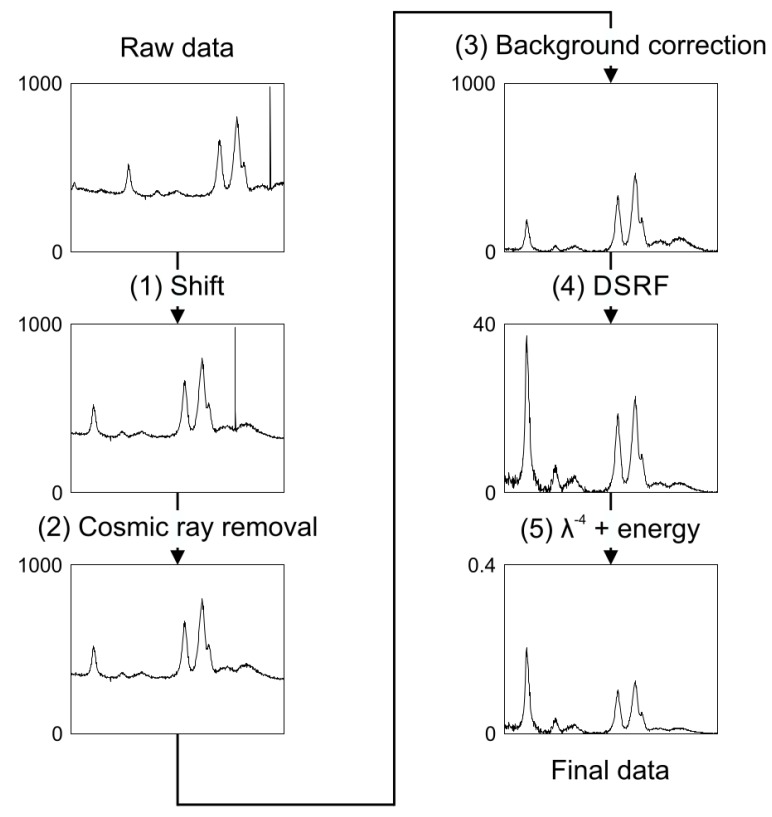
Post-processing of Raman spectral data. It includes renewal of spectral calibration if necessary, removal of artifacts such as cosmic rays or hot/cold pixels, background correction, and correction of the signal intensity for the device response, applied energy, and general spectral efficiency of scattering processes (reproduced with permission from [35]).

Figure 5 depicts the whole process of preparation of resonance Raman data from measured raw data for further analysis.

Occasionally, single steps in this data post-processing procedure may be left out. Background correction may not be necessary for data showing low fluorescence background. Correction for the device response function may be left out if the spectral response is fairly flat in the respective wavelength range and only the resonance profile but not the absolute resonance enhancement is of interest.

#### 2.1.3. Multivariate Spectral Analysis

The analysis of complex multi-component Raman spectra from biological samples requires multivariate analysis. Several methods is available to analyze and classify spectral data such as hierarchical and K-means cluster analysis, factor analysis, principal component analysis (PCA), discriminant analysis, or approaches such as support vector machines and neural networks [46,47].

Hierarchical cluster analysis (HCA) of data obtained by resonance Raman microspectroscopy is one of the most promising tools for rapid in vivo analysis of biological and medical samples allowing, for example, identification and assignment of cells, significant molecules, or bacterial strains in complex samples [48,49,50]. Single cell analysis is particularly challenging because intercellular variability can be high. Even spectra from the same chromophore may show considerable variance as different host environments affect the resonance Raman spectra.

To test HCA algorithms for their discrimination and association ability, we used a set of six different bacterial strains expressing three different chromophores: the carotenoids spheroidene and spirilloxanthin as similar, but not identical chromophores, and heme C as a distinctly different chromophore [51]. This experimental layout allowed us to test which of the algorithms are sensitive enough to handle spectra with a high intrinsic similarity in their distinctive spectral features due to a common resonant chromophore such as the carotenoids in the presence of highly dissimilar spectra originating from other chromophores. We compared seven algorithms (Single-Linkage (Nearest-Neighbor-Clustering), Complete-Linkage (Farthest-Neighbor-Clustering), Average-Linkage, Weighted-Average-Linkage, Centroid, Median, and Ward) for their ability to correctly cluster Raman spectra of single bacterial cells from the six bacterial strains. One important result from this study was that the signal-to-noise ratio (S/N) range is an important parameter for the successful clustering of Raman spectra. When this range is too large, spectra tend to be clustered by spectrum quality rather than by different chromophore and/or host organism. This finding implies an additional data post-processing step following the procedure presented in the last subsection, i.e., equalizing signal-to-noise to a range as appropriate for a given set of samples. Therefore, it can be necessary to artificially reduce S/N by introducing white noise to accomplish valid clustering results. As the Ward algorithm is quite popular in tasks similar to the study layout, we were surprised that based on our study only weighted-average-linkage can be fully recommended for clustering resonant Raman spectra of single cells independent of their chromophore. In the light of the aforementioned issue concerning signal-to-noise ratio, this clustering procedure excels because it optimizes spectrum quality with each clustering step through the reduction of random noise. The Ward algorithm only achieved an accuracy of less than 70% for spectra from the same chromophore, failing to maintain their spectral relation. Therefore, the results of our study clearly show that appropriate cluster algorithms need to be evaluated with respect to the specific application and caution is advised for unquestioned application of seemingly standard approaches.

**Figure 6:**
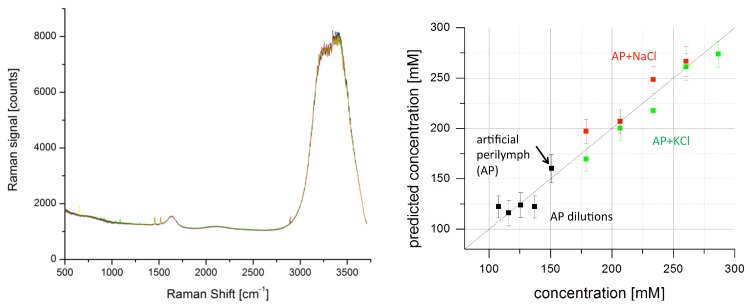
Raman spectra of samples of artificial perilymph that was either diluted or spiked with NaCl or KCl (**left**) and prediction of the small ion concentration by multivariate analysis based on the Raman spectra (**right**).

Multivariate analysis may also be applied to sets of Raman data for chemometric applications. A relatively simple application is the determination of the salt concentration in water. The concentration of small ions and their composition is crucial for many cell functions. For example, electrical communication involves sodium (Na${}^{+}$), potassium (K${}^{+}$), and calcium (Ca${}^{2+}$) and small ions provide the basis for setting up transmembrane potentials that are then used to power key processes such as ATP synthesis (involving hydrogen H${}^{+}$, Na${}^{+}$). They serve as cofactors in dictating protein function with entire classes of metalloproteins in processes ranging from photosynthesis to human respiration. Here, manganese (Mn${}^{2+}$), magnesium (Mg${}^{2+}$), and iron (Fe${}^{2+}$) play a crucial role. Moreover, small ions act as a stimulus for signaling and muscle action (Ca${}^{2+}$). Chloride (Cl${}^{-}$) generally acts as the main negatively charged counterpart. In inner ear fluids such as the endolymph or perilymph, deviation from the default concentration of small ions—especially of potassium—is connected to several forms of sensorineural hearing loss [52]. Therefore, being able to measure the small ion concentration non-invasively by optical means could support diagnosis in hearing research. Even though small ions cannot be Raman-active themselves, they can be measured indirectly by Raman spectroscopy via the water Raman signal [53]. The polar water molecules form cavities around the solved monatomic ions which affects especially the energy of their stretch vibrations showing in the broad range of coalescent Raman bands between 3000 and 3700 cm${}^{-1}$. Figure 6 shows Raman spectra of water containing artificial perilymph, dilutions thereof, and perilymph spiked with NaCl or KCl, i.e., water with a range of near physiological concentrations of small ions.

The base artificial perilymph contains 145 mM NaCl (sodium chloride), 2.7 mM KCl (potassium chloride), 2.0 mM MgSO${}_{4}$ (magnesium sulfate), 1.2 mM CaCl${}_{2}$ (calcium chloride), buffered by 5.0 mM HEPES and adjusted to pH 7.4 by NaOH (sodium hydroxide). Distilled water and NaCl or KCl were added to prepare dilutions or spiked samples. A model for prediction of the small ion concentration was developed using PCA and partial least squares (PLS) regression. As Figure 6 shows, the predictive power of the model is quite good for such a simple in vitro set of samples, at least for concentrations >150 mM. The trend of overestimation of sodium concentration and underestimation of potassium concentration seen in Figure 6 is not significant in this model given the number of samples but may point to a possibility for further discrimination with a more elaborate model and training data set. It must be noted though that in the body, the fluid would not only contain small ions but also other molecules such as proteins and peptides influencing water structure and the energies of the water stretch vibrations, so in vivo measurement of small ion concentration via the water Raman signal is expected to be much more difficult.

In a study of the violaxanthin cycle of algae [36], we were able show the potential and power of analysis of Raman spectral composition via PCA also for in vivo applications. The biological background and relevance of the violaxanthin cycle will be detailed in Section 2.3. Here, this example serves particularly well to depict how spectral principle components sometimes represent specific molecules.

**Figure 7:**
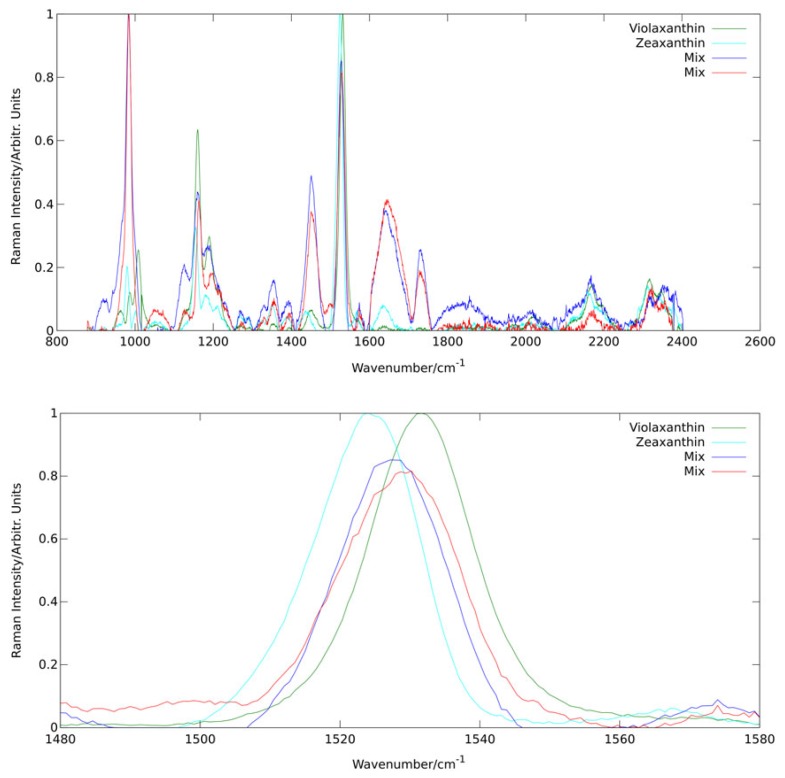
The first four principal components as analyzed by PCA from spectra of an algae culture (**top**). Analysis of major line positions such as the C=C stretch vibrations (**bottom**) sometimes allows assignment of certain molecules as the origin of the respective principal component (reproduced with permission from [36]).

Figure 7 shows the first four principal components derived by PCA from a dataset of more than 10,000 Raman spectra of a culture of the alga *dunaliella salina* excited at 473 nm. All four components are governed by spectral characteristics of carotenoids as these are (pre-)resonantly enhanced at this wavelength. Analysis of line positions allows even to assign specific carotenoids as the origin of the two first principal components. It is well known that zeaxanthin and violaxanthin are contained in *dunaliella salina* as they are interconverted by the alga in its violaxanthin cycle. The line positions of the $\nu_{1}$ band for the first two principal components are 1524 cm${}^{-1}$ and 1531 cm${}^{-1}$ matching well the values found by Ruban et al. [54] for zeaxanthin (1522 cm${}^{-1}$) and violaxanthin (1529 cm${}^{-1}$) in pyridine solution at 473 nm excitation. Pyridine is expected to reproduce line positions of carotenoids embedded in biological materials quite well [55]. The two mix components probably combine other carotenoids from the light harvesting complex.

### 2.2. Protein Analysis—From Molecule Function to Assessing Cell Systems

Today, proteomics—the study of the function of all expressed proteins—is a main research field within the life sciences. Hope is that it contributes at a direct level to a full description of cellular function [56]. The relevance of proteins for life is fundamental as genetic code is transferred into cellular function by means of proteins. Protein function and properties are determined by their amino acid sequence and their three-dimensional conformation or structure. Conventional approaches for protein identification and study of their 3D structure are mass spectrometry and X-ray crystallography. However, they do not work under physiological conditions. This is a severe drawback for many proteins as they tend to change conformation and lose function (degenerate) under non-physiological conditions, and consequently structures determined by x-ray crystallography may show a dysfunctional and not the native protein. Therefore, without depreciating the achievements of mass spectrometry and X-ray crystallography for proteomics, methods that can identify proteins and probe their three-dimensional structure and conformational changes under physiological conditions are highly esteemed. Nuclear magnetic resonance (NMR) [57,58], Raman spectroscopy [19,59,60], and recently cryo-electron microscopy [61,62] are the few available approaches here. Even though Raman spectroscopy cannot provide a tomographic image of the protein structure as can the other techniques, it has the advantage that it can be applied in living organisms allowing quasi-continuous measurement at a single sample and scales from molecular studies over cellular investigations to full-tissue studies.

#### 2.2.1. Connexin Hemichannel Gating

Connexins, or gap junction proteins, are structurally related transmembrane proteins that assemble to form channels between apposed cells in vertebrates. These channels are essential for many cell functions. Eight distinct human diseases have been definitively linked to germline mutations in connexin family members including the rather common non-syndromic sensorineural deafness, and various mechanisms to compensate connexin mutation or loss have evolved [63]. Within a cell, connexins assemble to form hemichannels that then couple to a hemichannel of the neighboring cell. To control transport through the gap junction channels, these hemichannels need to have open and closed states and to be switchable between these states by defined triggers. The human connexin 26 (hCx26) hemichannel is known for a complex, multi-facetted gating process. Several gate mechanisms have been identified in electrophysiological and biochemical studies [64]. For connexin 26, conformational changes are expected in plug and loop gating [65,66]. Both gating processes are yet to be fully understood. Besides, the gating process of hCx26 also contains a temperature-sensitive trigger component of yet unknown properties, changing the conductivity for small molecules from low to high upon exceeding a temperature of 23 °C [67].

**Figure 8:**
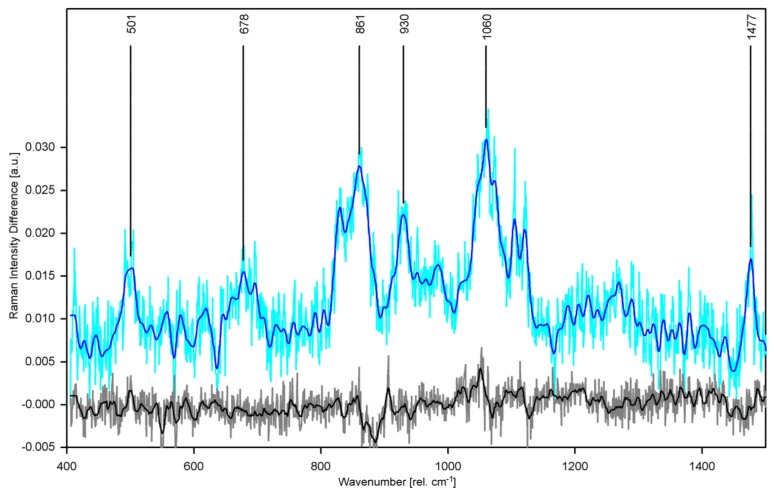
Difference spectrum of the connexin 26 hemichannel at 30 °C and 10 °C. The difference Raman signal points towards conformational changes for hCx 26 in Ca${}^{2+}$-buffered POPC (blue), whereas no conformational changes due to temperature change can be seen in the Raman spectra for hCx26 in POPC (black) (reproduced with permission from [15]).

Providing the first analysis of purified hCx26 protein and protein function based on Raman spectroscopy, we studied structural differences in the hCx26 hemichannel at temperatures above and below the switching temperature of 23 °C [15]. For this purpose, we recorded and analyzed high-precision Raman spectra of purified hCx26 at 10 °C and 30 °C by confocal Raman microscopy. Raman spectra for various experimental conditions (temperature, buffer) were compared by pairwise calculation of difference spectra to spot spectral differences indicating conformational change. We found that the Raman spectrum of hCx26 in Ca${}^{2+}$-buffered POPC at 10 °C significantly differs from all others (see Figure 8).

Raman bands appearing in the difference spectrum mark features that are present in hCx26 at 30 °C but have disappeared at 10 °C. Analysis of possible origins of the changed Raman spectrum within the molecule identified specific amino acids (tyrosine, histidine, cysteine) and the α-helical backbone. These results confirm that specific protein structures (TM1/EL1 parahelix and probably the TM4 transmembrane helix) are involved in the gating process responsible for fully closing the hemichannel. Besides, we found an indicator for the fully closed state of the hemichannel (absence of the Raman signal of the imidazole group of histidine) which may also indicate that the plug domain of hCx26 is also involved in the temperature-sensing gating mechanism. These structural findings contain important new information elucidating the gating mechanisms of hCx26 that were not achieved by application of other methods in other groups before.

#### 2.2.2. Bacteria Identification

Besides the (functional) analysis of proteins on the molecular level, Raman spectroscopy is also applied on a cellular or macroscopic scale. These larger scale applications include diagnostic purposes, for example as a tool for cancer diagnosis [18,68], as well as identification of microorganisms [16,69,70] and their cohabitation in biofilms [71]. The spectral features in the Raman spectra from such targets usually originate from a multitude of molecular sources such as proteins, lipids, or chromophores such as carotenoids. Such complex Raman spectra may be used to identify microorganisms, cells, or pathologic changes, etc. even without identification of the single components involved. However, identification of single significant molecular components within the spectra opens the door to more fundamental investigations such as identification of molecular biomarkers or possible insights into the phylogeny of bacteria.

We have applied Raman microspectroscopy to study bacteria in their native environment, the biofilm, taking advantage of (pre-)resonance enhancement of proteins and small molecules [72,73]. Raman microspectroscopy allows identification and imaging of bacteria in biofilms also providing information about cell morphology and arrangement or diversity of different microcolonies. Other biofilm components such as minerals or microplastics generally appear as microparticles and present quite pure and strong Raman spectra even without signal enhancement or appear as dark areas in the Raman images.

**Figure 9:**
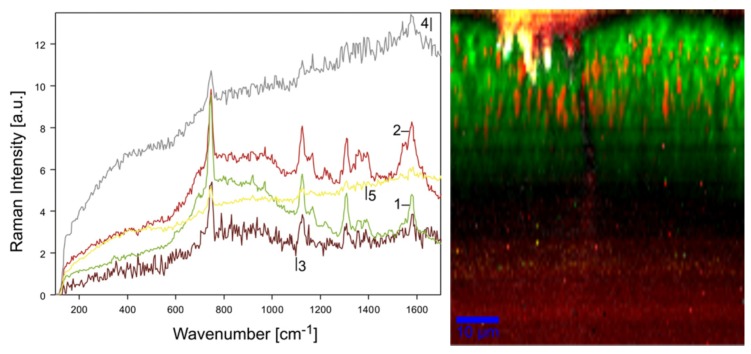
(Resonant) Raman spectra (**left**) and outer layer of a biofilm granule (**right**) showing a dense multilayer of microcolonies crossed centrally by a vertical canal. The color of the spectra also provide the color-code of the image; bacterial seed fingerprint type-I identified as *nitrosomonas communis* (1, green), bacterial seed fingerprint type-II, probably *nitrosomonas europaea*, (2, red), and cytochrome c spectrum 80 μm below the surface (3, brown) with too low S/N to be assigned to a specific species. The spectra 4 and 5 denote weak and weakest autofluorescence spectra depicted in the image as white and yellow areas (reproduced with permission from [72]).

In [72], we studied biofilm composition in flocs from sequencing batch reactors (SBRs) mapping their development over three months. Figure 9 shows an xz-image of a granule sampled after one month of operation from one of the SBRs. Two major bacteria fingerprints were identified in the seed material for the SBRs, coming from a reactor using a very efficient type of wastewater treatment.

The corresponding bacteria, type-I and type-II, and their co-development in the biofilm were traced by sampling of biofilm granules over time. As shown in Figure 9 confocal Raman microspectroscopy can visualize the organization, i.e., co-localization, of bacteria in the living biofilm. Type-I bacteria form the bulk of the biofilm while type-II bacteria nest within and are not found in direct contact with water at the vertical canal or the surface of the biofilm. The two types of bacterial colonies have very similar appearance so that they could only be discriminated by their Raman fingerprints. The main molecular contribution to these fingerprints comes from cytochrome c. Cytochrome c is a particularly interesting protein because its amino acid sequence and structure are highly conserved across the spectrum of species. All cytochrome c proteins contain a characteristic amino acid motif that binds heme, and heme is resonantly enhanced at 532 nm, a standard excitation wavelength in confocal laser microscopy, which is also used in our confocal Raman microscope. The protein shell around the heme differs for cytochrome c proteins in different organism affecting the vibronic energy levels and, thus, the Raman fingerprint of the heme core. Even though these spectral differences are hard to spot and appreciate by eye (compare Figure 9, spectra 1 and 2), multivariate analysis as presented in Section 2.1.3 can discriminate these spectra. Moreover, comparison with reference data from cultured strains of bacteria allowed identification of type-I bacteria recorded from the seed mass as *nitrosomonas communis* Nm-02 with 94% certainty. Spectrum comparison between type-II bacteria and the other available reference spectra of cultured bacteria point to a *nitrosomonas europaea*. *N. europaea* references (Nm-50, Nm-53) showed a spectral similarity of approximately 80% to the type-II spectra, indicating a different strain or a phenotype variant. This assessment is based on our experience with assignment of pure bacteria cultures to their respective species or strain via resonant Raman spectra recorded from individual cells. Here, bacteria can be identified with 96% certainty when compared to spectra of the same strain and with 84% certainty when compared to spectra of a different strain of the same species.

Other chromophores such as carotenoids expressed by bacteria also help to narrow down the pool of possible candidates behind a Raman fingerprint of bacterial cells even though it is not as specific as the cytochrome c signal [72].

### 2.3. Carotenoids

Carotenoids are yellow to red organic pigments that are primarily produced in plants. Their function in nature is mainly based on their characteristics as one of the major chromophores in this spectral range and their antioxidant capacity. In plants and algae, they serve in light absorption and energy transfer in photosynthesis and protect other pigments from photodamage. In humans, they play a key role in vision. Carotenoids have been extensively studied spectroscopically—for example to elucidate their critical role and function in light absorption and energy transfer in photosynthesis. In this section, we discuss important experimental and analytical issues for a valid interpretation of resonance Raman data of carotenoids (Section 2.3.1), which apply similarly to other resonantly enhanced Raman signals, and show how resonance Raman spectroscopy can be applied to watch carotenoid kinetics in the living organism (Section 2.3.2).

#### 2.3.1. Solvent Effects

As pointed out before, Raman resonance and absorption spectra are strongly connected. Therefore, knowledge about the absorption characteristics of a given sample usually allows good guesses at excitation ranges for optimal resonance enhancement. Carotenoids absorb in the blue to green wavelength range, often with a pronounced vibronic structure, and typically gain additional UV absorption when the hydrocarbon backbone is in *cis*-conformation instead of the *all-trans* form. The absorption properties of carotenoids heavily depend on their molecular environment. In carotenoids which contain carbonyl functional groups in conjugation with the carbon-carbon π-electron system, vibronic structure decreases dramatically when the molecules are dissolved in more polar solvents. This is accompanied by considerable spectral broadening [74]. Besides, the polarizability of the solvent may cause considerable shifts of the whole absorption band: The higher the refractive index *n* of the solvent, the larger the bathochromic shift.

**Figure 10:**
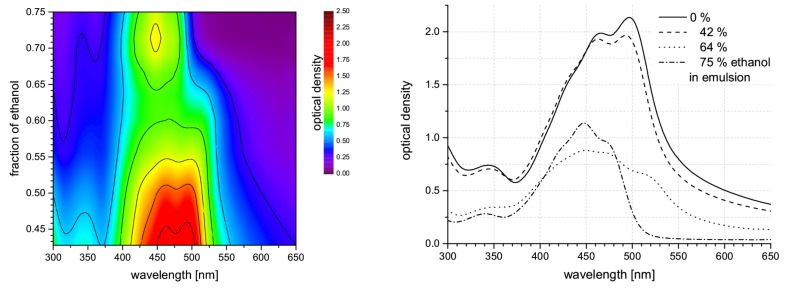
Absorption spectra of β-carotene emulsion depending on the ratio of ethanol and water. Spectra were scaled to the same β-carotene concentration assuming linear dependence of optical density on the concentration. The spectral map (**left**) quasi-continuously shows the transformation of the absorption characteristics of β-carotene emulsion in aqueous solution from 42% ethanol to 75% ethanol content. Three spectra from characteristic points of this series of measurements and an additional spectrum of β-carotene emulsion in pure water (from a different stock solution) are compiled in the graph on the **right** (reproduced with permission from [76]).

For application of resonance Raman spectroscopy in vivo, it is important to foster awareness of these effects. As mentioned before (Section 2.1.3), solution of carotenoids in pyridine is expected to reproduce spectral characteristics of carotenoids embedded in biological materials quite well [55]. Pyridine causes a bathochromic shift of carotenoid absorption spectra by 18–24 nm compared to the spectral positions in ethanol for example [75]. Against this background, we studied solvent effects on carotenoid absorption and resonance Raman spectra. Figure 10 shows the transition of the β-carotene absorption spectrum between characteristic points crucial for considerations concerning in situ measurements.

An emulsion of β-carotene (Altratene 5% EM, 5.9% emulsion) in 100 mg water was diluted by ethanol. At 42% ethanol content, the β-carotene absorption spectrum still shows characteristic features of β-carotene in emulsion, a lipid environment. Absorption maxima of β-carotene appear at approximately 466 nm and 497 nm. With increasing ethanol content, the β-carotene spectrum gains features of aggregated β-carotene which is most clearly seen in a new peak or shoulder at approximately 518 nm. The classic β-carotene absorption band around 450 nm starts to appear as well in this solvent environment. Finally, the spectrum from β-carotene emulsion in 75% ethanol already shows the characteristics of β-carotene in pure ethanol—which may be called the standard spectrum—quite clearly.

**Figure 11:**
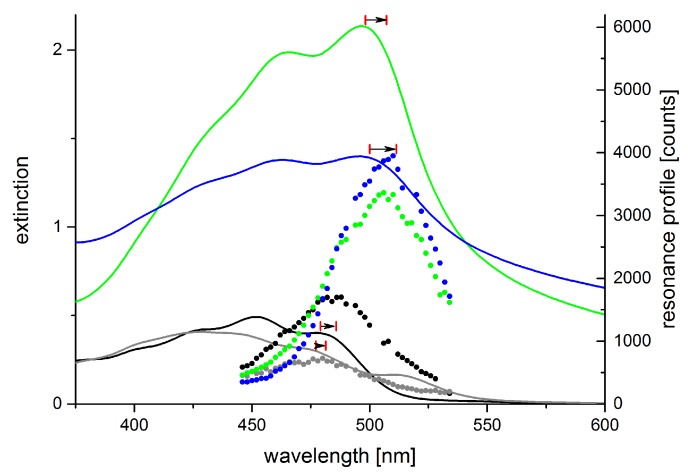
Absorption spectrum (solid lines) and resonance profiles of the strongest carotenoid Raman peak at ca. 1528 cm${}^{-1}$ (C=C stretch) (dots) for β-carotene in different molecular environments. From top to bottom, the curves show resonance maps for 64 mg Altratene EM5% emulsion in 100 mL of water (green), a hydrogel stained with 11 mg Altratene EM5% emulsion in 100 mL of aqueous solution containing <12% ethanol (blue), and ethanol solutions with 20% (black) or 40% (gray) water content. Distance marks (arrows) at each 0-0 transition peak indicate the spectral shift between absorption and resonance maximum (reproduced with permission from (reproduced with permission from [76]).

These spectral changes are highly important for considerations concerning *in situ* studies on plants, animals, or humans, as absorption spectra of β-carotene in organic solvents such as ethanol are often consulted to derive expected resonance conditions whereas, physiologically, a lipid environment similar to the emulsion is to be expected for water-insoluble carotenoids. We studied the effect of these spectral shifts and changes on the Raman resonance behavior of β-carotene in artificial samples such as liquid or hydrogel sample for reference. As expected, the Raman resonances generally shift with the spectral origin of the absorption spectrum, i.e., the longest wavelength absorption peak (see Figure 11).

The resonance maximum of Raman scattering is always slightly red-shifted compared to the 0–0 transition absorption maximum (peak positions were evaluated by linear multi-peak fitting with Voigt-profiles). Aggregates of carotenoids are present in the sample of β-carotene dissolved in ethanol containing 40% water as revealed by the additional long-wave peak at 518 nm. The Raman resonance, however, still follows the absorption peak at 477 nm. Even though the β-carotene concentration used in the hydrogel sample from β-carotene emulsion is only ca. 20% higher than that in the 20% water-ethanol solution, Raman intensities more than double. The increase in absorbance, however, is even larger than the resonance increase of the Raman signals. Of course, resonance Raman spectral intensities generally suffer from self-absorption. For the β-carotene samples derived from emulsion, the spectral extinction in a 10 mm cuvette is above 1 and 2, respectively, in the resonance range. Accordingly, Raman signals are significantly attenuated in these samples. Therefore, if molecules are to be identified by their resonance excitation profile as presented in Section 2.3.2, solvent conditions must be considered carefully. Especially if quantification of carotenoids is intended, the dependence of absorbance on the solvent and non-linearities in the concentration-absorbance relation need to be taken into account [77,78].

**Figure 12:**
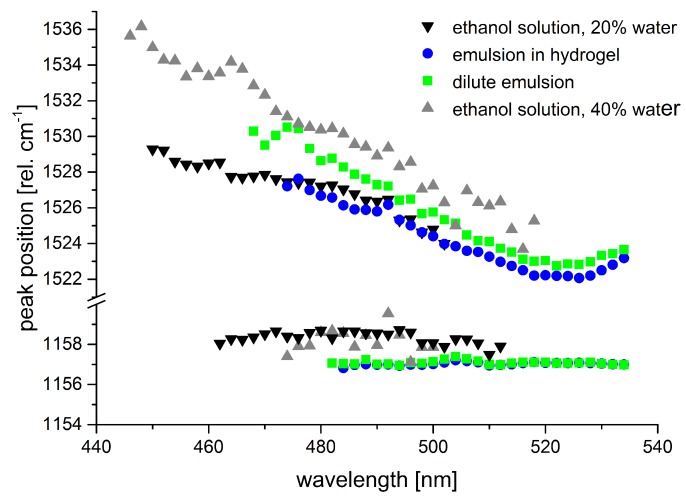
Peak positions of the C-C (1157 cm${}^{-1}$) and C=C (around 1528 cm${}^{-1}$) vibrations of β-carotene in different molecular environments, i.e., for the same samples as in Figure 11. Please note that all spectra were spectrally calibrated to the C-C vibration at a Raman shift of 1157 cm${}^{-1}$ due to lack of a common external standard. Therefore, peak-to-peak distances but not absolute peak positions are reliable (reproduced with permission from [76]).

Given that the Raman fingerprint of a molecule is composed of the single Raman peak positions of characteristic vibrations of the molecule, we also studied the dependence of Raman peak positions on the excitation wavelength and on the solvent conditions. Figure 12 shows the peak positions of the Raman lines originating from the C=C and C-C stretch vibrations of β-carotene for the samples shown in Figure 11.

Tschirner et al. [79] found a wavelength-dependent variation of the peak maximum of the C=C stretch vibration for β-carotene in dichloromethane that they attributed to different relative resonances of the two underlying modes. According to their experimental data, the peak maximum shifts from 1525 cm${}^{-1}$ at 1064 nm to 1521 cm${}^{-1}$ at around 514 nm and up again to 1525 cm${}^{-1}$ at 465 nm. We observed similar albeit larger peak shifts for the C=C Raman band in our data subject to the restriction that our data was spectrally calibrated to the C-C stretch vibration of β-carotene as no common external standard was available for calibration. The samples from emulsion show a minimum in Raman peak position at excitation around 523 nm matching the observations of [79]. Our data from the samples of β-carotene in ethanol-water mixtures does not show such a minimal Raman shift, but this is probably due to the fact that peak positions were not determined in the wavelength range where the minimum is expected as signal-to-noise ratio was too low. As peak positions still rise towards the short-wave end of the available data, absolute values for the maximal peak positions cannot be clarified from our data. Still, we can state a peak shift of at least 5–7 cm${}^{-1}$ within the resonance range for the emulsion-based samples and 4 to more than 10 cm${}^{-1}$ for the ethanol-water solutions.

The solvent effects described here for β-carotene may be transferred in a similar manner to many other carotenoids. This knowledge is crucial to a valid analysis of carotenoids in tissues or plants and of special importance when quantification of specific carotenoids is intended beside their identification. The dependence of the Raman peak position associated with the C=C stretch vibration on the excitation wavelength is the parameter that is least likely to cause misinterpretation of in situ measurements. Usually, excitation at single fixed frequencies from standard, i.e., common, laser systems is applied in these measurements [80,81]. Therefore, reference spectra are available. Besides, due to similar resonance conditions, the carotenoid signal from biological samples hardly ever originates from a single carotenoid, but from several carotenoids with quite similar Raman fingerprints causing line broadening in the Raman signal.

The solvent-dependent resonance shift, however, should be carefully considered in the quantification of single carotenoid components. This is especially true when mixtures of carotenoids as found in human skin or plants are assessed. For example, the groups of Werner Gellermann and Jürgen Lademann propose an elegant approach deriving β-carotene and lycopene concentration from the Raman spectra of human skin even taking into consideration the effect of different skin colors by reflectance measurements [82,83]. They use excitation at 488 nm and 514.5 nm and derive the relative excitation efficiency or scattering cross-sections, respectively, from measurements on carotenoid solutions in acetone and ethanol, respectively. Probably, using carotenoids in pyridine or lipids for reference could further improve the accuracy of the quantitative measurement based on their approach.

#### 2.3.2. Carotenoid Transitions in Algae

Photoautotrophic organisms such as plants collect light energy in the so-called antenna complexes for later carbon fixation. Oxygenic organisms use accessory pigments to transfer energy to photo system chlorophyll. One important class of these accessory pigments are the carotenoids. The direction of energy transfer depends on the individual energy levels of the involved pigments and is tailored to efficiently collect light energy throughout the visible wavelength range (with a minimum in the green range). In the case of excess light, the parameters of Förster resonance energy transfer (FRET) are used by some photoautotrophic organisms for regulation of energy flow to prevent irreversible damage to the photosystem. The two xantophylls (i.e., oxygen containing carotenoids) violaxanthin and zeaxanthin are well known to provide a regulatory function called the violaxanthin cycle [84]. Violaxanthin transfers absorbed energy to chlorophyll in the light harvesting complex and hence to the photochemical reaction center. In a reversible enzymatic reaction [85] plants transform violaxanthin over the intermediate step antheraxanthin into zeaxanthin within a timescale of minutes to a few hours. Zeaxanthin possesses 11 conjugated double bonds as compared to nine in violaxanthin resulting in a larger π-electron system and thus the energy of the first excited singlet state is lower than in violaxanthin—too low for efficient energy transfer to chlorophyll a. So, zeaxanthin acts as an energy trap at the violaxanthin position in the light harvesting complex and removes excess energy via heat dissipation.

So far, it was not possible to monitor the carotenoid interconversion and, thus, the violaxanthin cycle in situ. Knowledge about involved carotenoids, cycle kinetics, etc. was mainly gathered by high-performance liquid chromatography (HPLC) making consecutive measurements on living organisms impossible.

##### Violaxanthin Cycle Kinetics via PCA of Raman Spectra

**Figure 13:**
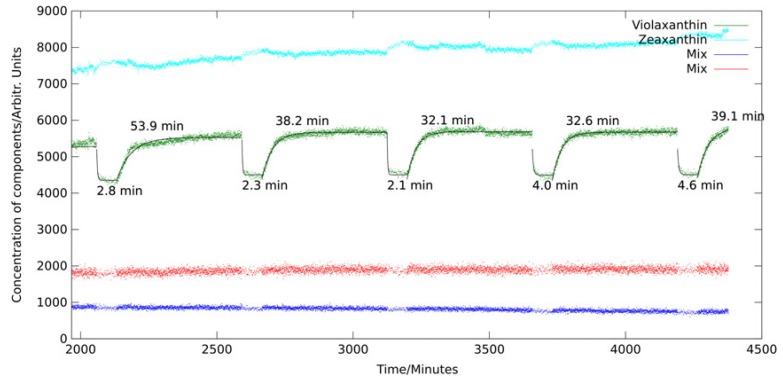
Kinetics of the light-to-dark and dark-to-light transition response of violaxanthin (green) and zeaxanthin (cyan). The upper level of the violaxanthin trace marks the dark-adapted state, the lower level the state at excess light conditions. The zeaxanthin trace shows the complementary kinetics, albeit weakly. The two nearly constant traces (red and blue) represent chlorophylls and carotenoids not directly involved in the violaxanthin cycle reactions. Consequently, those do not show variable concentration in reaction to light stress (reproduced with permission from [36]).

In Koch et al. [36], we presented the first in vivo measurements of violaxanthin cycle kinetics. The alga *dunaliella salina* was intermittently exposed to dark periods and photostress by high-power LED illumination. Resonance Raman spectra were continuously acquired via a fiber bundle submerged in the stirred algae culture. PCA (see Section 2.1.3) was applied to quantify the involved pigments. Figure 13 shows the kinetics of the principal components assigned to violaxanthin, zeaxanthin and two other principal components describing mixed pigment groups of *d. salina* mainly consisting of chlorophylls and other carotenoids.

Signal enhancement for chlorophyll and violaxanthin is stronger than for zeaxanthin as the excitation wavelength better matches the 0–0 transition of violaxanthin than the 0–0 transition of zeaxanthin. This leads to a lower signal-to-noise ratio in the zeaxanthin trace. Time constants for transitions were determined by exponential fitting to the violaxanthin trace yielding between 39 and 46 min for the light-to-dark transition depending on experimental conditions and 3.2 min for the dark-to-light transition. These findings are in good agreement with data obtained by HPLC analysis in different plants [85,86,87].

A few hours after the light stress, we could also observe a decrease of both violaxanthin and zeaxanthin in longer periods of darkness corresponding to a reduction of the violaxanthin cycle regulation capacity. Pool size adjustments of the carotenoids involved in the violaxanthin cycle are known, but on a larger time scale of a few days, as reported by Nichelmann et al. [88].

##### Carotenoid Transition in Stress Reactions of Algae via Analysis of Raman Resonance Profiles

As shown in the previous section, PCA is a powerful tool for in situ investigation of pigment kinetics via Raman spectroscopy. Looking at the spectral differences in the principal components assigned to the two main involved carotenoids violaxanthin and zeaxanthin, it is obvious that this approach benefits strongly from high spectral resolution. PCA of data from a low-resolution spectrograph for example would not achieve the same discriminative power or even fail to depict the interconversion at all. Resonance Raman spectroscopy, however, provides another means of chromophore discrimination besides the Raman fingerprint: the resonance behavior. Resonance conditions often differ even for relatively similar pigments. This effect is particularly strong in carotenoids which all share a common structure. They are tetraterpenoids, i.e., they were produced from eight isoprene molecules and contain 40 carbon atoms. Still, the length of the conjugated carbon chain may differ for different carotenoids resulting in different absorption maxima in the blue to green range.

**Figure 14:**
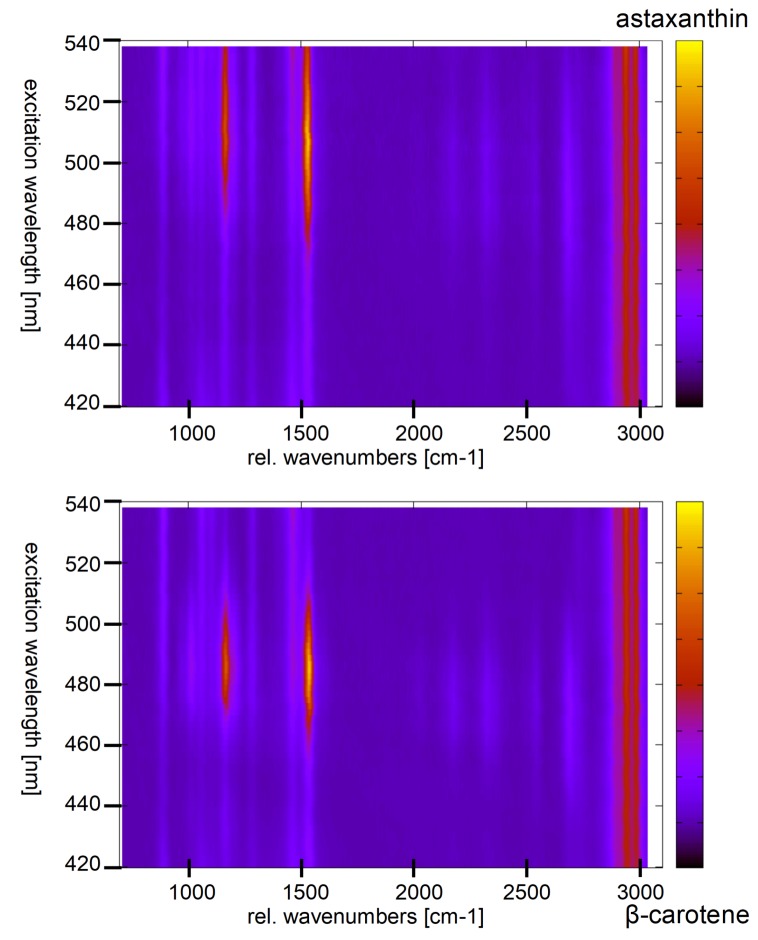
Resonance maps of astaxanthin (**top**) and β-carotene (**bottom**) solved in ethanol. The triple-band around 2900 cm${}^{-1}$ showing constant intensity over the whole excitation range originates from ethanol as do all constant lines in the fingerprint region. All lines with varying intensity originate from the carotenoids. Above 2000 cm${}^{-1}$ variable Raman lines are overtones and combination bands (reproduced with permission from [91]).

Green algae generally have high contents of α- and β-carotene, violaxanthin, neoxanthin, and lutein [89]. Some of them can produce secondary carotenoids or other pigments under stress. For example, *haematococcus pluvialis* accumulates astaxanthin under starvation conditions. It amounts up to 4% cell dry weight (2.6 g L${}^{-1}$) accounting for more than 99% of the total carotenoid content in stressed *haematococcus pluvialis* cysts [90]. The maximum of resonance enhancement of the carotenoid lines is expected to change according to the carotenoid composition of a sample reflecting their different absorption characteristics. Figure 14 shows the resonance maps of astaxanthin and β-carotene in solution to illustrate the resonance shift between two carotenes involved.

**Figure 15:**
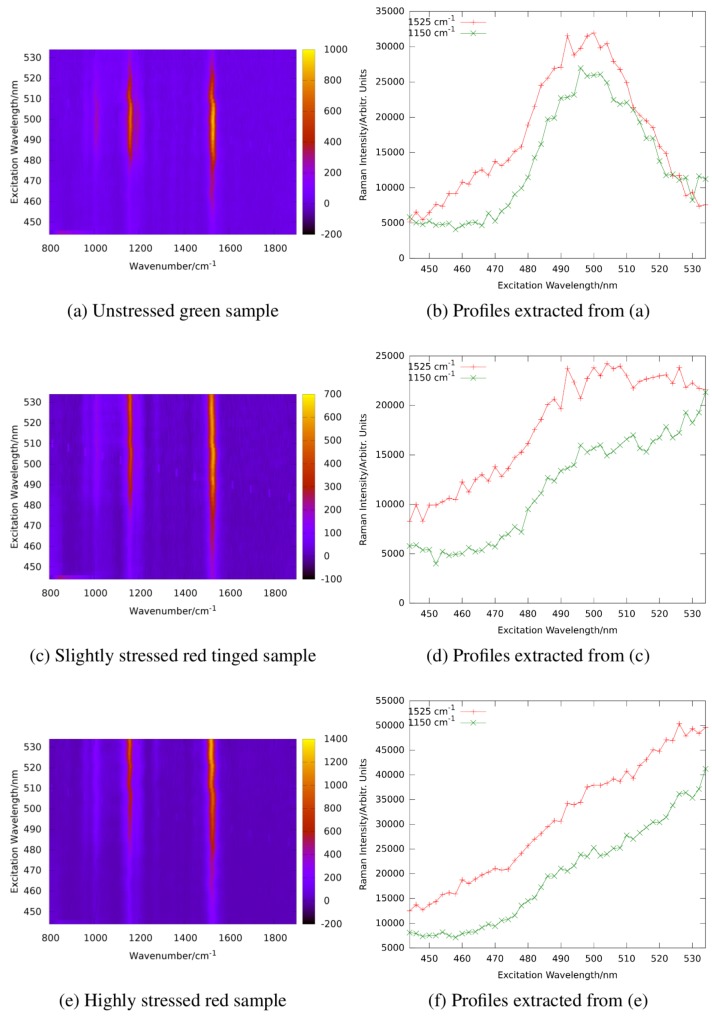
Resonance Raman maps (**left**) and corresponding resonance profiles (**right**) of *haematococcus pluvialis* at different stress levels: unstressed (**upper** row), slightly stressed (**middle** row) and strongly stressed (**bottom** row) (reproduced with permission from [92]).

In ethanol, the resonance maximum of lutein is at excitation around 476 nm and α-carotene, violaxanthin, and neoxanthin are expected to show maximum resonance well below 500 nm as well. We analyzed the resonance Raman spectra of *haematococcus pluvialis* and two other green algae at different levels of starvation caused by nutrient deficiency [92]. As indicated by Figure 14, a significant red shift of the resonance maxima of carotenoid lines is expected during transition of the algae from the unstressed green state to formation of stressed red cysts. Figure 15 shows resonance Raman maps and profiles for the unstressed, an intermediate, and the stressed state.

The resonance maximum of the carotenoid lines is continuously red-shifted with increasing nutrient stress and starvation as expected for increasing concentrations of astaxanthin in the algae cells. It should not go unnoticed that resonance maxima of the stressed algae sample shift even beyond 530 nm which is beyond those of astaxanthin in ethanol solution shown in Figure 14. This is due to the different molecular environment; in the algae cells, the carotenoids are embedded in a lipid environment leading to red-shifted resonance compared to the rather apolar ethanol solution as already expanded upon in Section 2.3.1.

In summary, our work concerning resonance Raman spectroscopy on carotenoid transitions in algae resulted in a new and promising tool for in situ studies allowing for the first time quasi-continuous observation of the carotenoid transition kinetics in photoautotroph organisms.

Therefore, Raman spectroscopy excels in providing highly specific molecular information on biological samples. Measurements are non-invasive and can be carried out in situ. However, in general, imaging or spatially resolved measurement is slow because of the low quantum yield of the Raman effect and it is restricted to superficial volumes because of the considerable optical attenuation in biological material. Besides, the measurement of absolute quantities such as concentration of a target molecule in the tissue is difficult as at least the spectral attenuation within the individual tissue or sample needs to be known for this task, but can hardly be measured in vivo. Consequently, it is highly rewarding to combine Raman spectroscopy with other (optical) modalities for mutual benefit. In the following Section 3, we present optoacoustics as a complementary 3D imaging and measurement method. While optoacoustics is a well-known method for non-invasive imaging of living tissue, we will rather address the issue of the unknown optical properties of individual tissue here and focus on deriving spectrally and spatially resolved optical absorption coefficients from optoacoustic data.

## 3. Optoacoustics

Optoacoustics is a hybrid method combining optical contrast with acoustic signal propagation. As biological samples generally show strong optical attenuation, using optoacoustics, optical information can be collected from much deeper inside a sample compared to purely optical methods. This advantage has made optoacoustics highly attractive for in vivo biomedical application in recent years—not without good reason did Li and Wang [93] call photoacoustics “the fastest growing new biomedical method, with clinical applications on the way” in 2009. It has been promoted as a future valuable tool for cancer detection and diagnosis, tumor characterization and treatment guidance [94], and even attempts at obtaining absolute chromophore concentrations from photoacoustic images have been made [95].

It needs to be pointed out that optoacoustics (or photoacoustics, which is often used synonymously) has been primarily applied for imaging and tomography. An alternative use, which has mainly been followed in the work presented here, is its application as a quantitative measurement tool for optical (and acoustic) properties of the target. A few attempts can be found in the literature, see [95] for a review, but there is no single comprehensive approach mainly because the theoretical description of the problem is under-determined (see below). Rather, approaches focus on certain conditions allowing specific assumptions or simplifications that lead to good approximation of a target property in this specific case.

**Figure 16:**
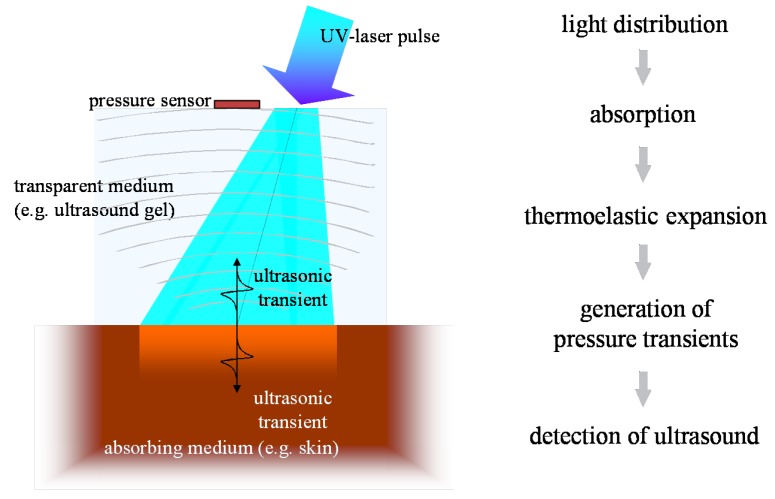
Principle of optoacoustics: the process of thermo-optical excitation of ultrasound (reproduced with permission from [96]).

The mechanism behind optoacoustics is the absorption of radiation energy in matter, its transfer to heat and pressure and the release of a resulting transient stress wave. Figure 16 sketches the underlying process: thermo-optical excitation of ultrasound.

Radiation propagates in the sample according to its optical properties. Eventually, all the radiation energy is absorbed and transferred to heat—ideally instantaneously and with radiationless transitions. This leads to a location dependent pressure rise in the sample, which is isotropically released in an ultrasonic transient. If the incident light pulses are sufficiently short—typically in the low nanosecond range—the profile of the stress transient reproduces the distribution of heat sources in the sample and consequently, the light distribution in the sample can be deduced. The traveling pressure transient then is subject to a variety of acoustic influences. Dissipation, nonlinearity effects, and acoustic diffraction may alter the profile of the initial stress wave depending on the acoustic properties of the propagation medium as well as the geometry of the sources, the detector, and their relative position. A comprehensive theoretical description of optoacoustics can be found in [97]. Condensed overviews providing some additional insights into the mechanisms of optoacoustics are set out in [98,99,100,101,102].

Even though the optoacoustic signal of in vivo samples can be measured anywhere outside the target if acoustic coupling is ensured, illumination and acoustic detection are usually positioned at the same side of the object to minimize losses. This detection mode is called backward mode. Measurement through a target object is generally also possible. This so-called forward mode is often used for thin samples but as it is of little relevance for in situ application, it will not be considered here.

### 3.1. Calculation of Optical Properties from Optoacoustic Measurements—Forward Solution

Generally, two approaches may be followed to measure optical properties by means of optoacoustics: a fitting procedure or solving the inverse problem. Both approaches become increasingly difficult or even under-determined with increasing complexity of the sample. Complexity in this case refers to the localization dependent variability of the absorption and scattering properties.

A very simple fitting procedure was applied to retrieve the (homogeneous) absorption coefficients of human skin in vivo in the UVB and UVA-II range (290–341 nm) [103,104,105]. The free optical path in this range is very low, only an order of magnitude larger than the spatial resolution limit of optoacoustics, and light distribution is governed by absorption rather than scattering, so assuming homogeneous optical properties is feasible for the fitting procedure in this case. The thermo-optical excitation of ultrasound by UV light in skin under the geometric conditions of the experimental setup used in these experiments can be described as

(1) $$\begin{array}{ccc} p(z,\tau ,r_{⊥}=0) & = & 0.57\left(p_{0}\left(\tau \right)-\int_{-\infty }^{\tau }\omega_{D}e^{-\omega_{D}\left(\tau -t\right)}p_{0}\left(t\right)dt\right) \end{array}$$

(2) $$\begin{array}{ccc} p(z=0,\tau ,r_{⊥}=0) & = & \rho_{0}c_{0}\frac{1}{1+N}v_{r}\left(\tau \right) \end{array}$$

(3) $$\begin{array}{ccc} v_{r}\left(z=c_{0}\tau \right) & = & \frac{\beta I_{0}}{2\rho_{0}c_{p}}\mu_{a}\left(c_{0}\tau \right)c_{0}e^{-\int_{0}^{c_{0}\tau }\mu_{a}\left(\xi \right)d\xi }\int_{-\infty }^{\infty }f\left(\tau \right)d\tau \end{array}$$

The pressure distribution $p(z,\tau ,r_{⊥}=0)$ measured at the retarded time τ at a detector that is placed centrally above the light distribution within the sample depends on the initial pressure distribution $p_{0}$, the acoustic transmission on the way from the sample to the detector (0.57 in this case), and a diffraction term for the acoustic wave which is described in terms of the characteristic diffraction frequency $\omega_{D}=\frac{2zc}{a^{2}}$. Diffraction leads to a deformation of the ultrasound transient propagating at the speed of sound *c*. At small distances *z* from the sample and/or large radii *a* of the illuminated area, diffraction is negligible, and the initial pressure distribution is reproduced at the detector. Together, the characteristic optoacoustic diffraction frequency $\omega_{D}$ and the characteristic frequency of the optoacoustic spectrum $\omega_{a}=\mu_{a}c$ can be used to define the acoustic near-field and far-field via the diffraction parameter $D=\omega_{D}/\omega_{a}$ as D<1 and D>1, respectively. The optoacoustic signal scales linearly with the incident intensity $I_{0}$ and the absorption coefficient $\mu_{a}$. The thermal coefficient of volume expansion of the sample is denoted as β while $\rho_{0}$ marks the average density of the sample and $c_{p}$ the specific heat at constant pressure. Sound velocities *c* of the tissue ($c_{0}$) or of the transparent medium ($c_{tr}$) used to acoustically connect skin and detector apply in the respective zones. If possible, the sound velocities of the sample and of the transparent medium are matched to avoid reflections. Ultrasound gel may be used for example.

**Figure 17:**
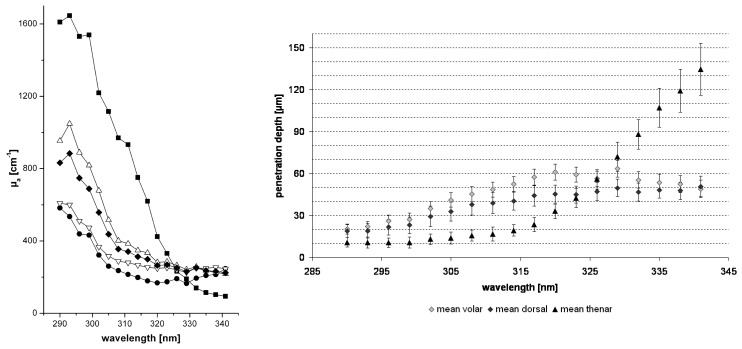
**Left**: Mean absorption spectra at the ball of the thumb (■, n = 20), and on the inner (●, n = 18) and outer (⧫, n = 20) side of the forearm. Additionally, mean absorption spectra at the outer side of the forearm are split up into a group reporting high (△, n = 12) and low (▽, n = 8) exposure to solar radiation, respectively (reproduced with permission from [105]). **Right**: Penetration depths (1/e-level) of UV radiation in human skin. Three different skin sites are shown: the inner (volar) side of the forearm, where pigmentation is comparably low, the outer (dorsal) side of the forearm that is generally more exposed to solar radiation resulting in stronger pigmentation and the ball of the thumb (thenar). Error bars mark 90% confidence intervals (reproduced with permission from [104]).

By aid of a transfer function describing the effect of the detector response on the optoacoustic signal, the absorption coefficient of human skin in vivo could be calculated with an uncertainty in the order of 20% by a fitting procedure weighting the amplitude of the main optoacoustic peak as the most important parameter as this amplitude is directly proportional to the absorption coefficient $\mu_{a}$. Our work provided the first and only in vivo data of human skin in the ultraviolet range available even today. Figure 17 shows data obtained from the study including n = 20 volunteers.

**Figure 18:**
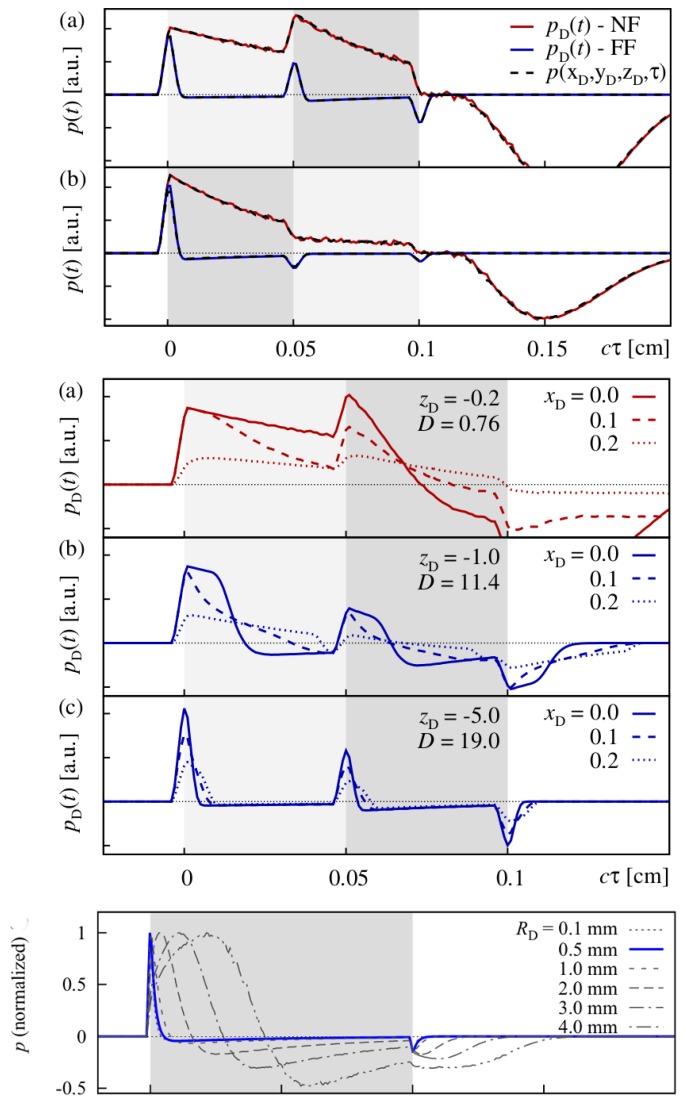
Influence of geometrical factors in the detector setup on measured optoacoustic signal. Gray shadings mark optically absorbing layers of different relative strength. The **top panel** shows a comparison of different solvers for the optoacoustic problem for layered media in two reversed layer scenarios (a and b). The measured signal $p_{D}\left(t\right)$ is shown in the acoustic near-field (NF) at a distance *z* of 0.04 cm between sample and detector and for far-field (FF) conditions at *z* = 4.0 cm (reproduced with permission from [106]). Calculations refer to detection on the illumination beam axis while the **middle panel** shows how the signal deforms upon displacement *x* of the detector off the beam axis for NF (a) to FF (c) conditions (reproduced with permission from [106]). The bottom panel shows the effect of the detector size (detector radius $R_{D}$) for an optoacoustic signal in the far field (reproduced with permission from [107]).

Besides the acousto-electrical transfer function that also includes, for example, the frequency response of the transducer, the measured optoacoustic signal is also strongly affected by the detection geometry. Figure 18 shows how the optoacoustic signal transforms depending on the distance of the detector from the surface of the initial pressure profile, the radial displacement of the detector from the center of illumination, and the detector size. The data in Figure 18 was produced by a more sophisticated numerical model that we developed to address the inverse solution of the optoacoustic problem [106,107]—more details concerning this approach will be presented in Section 3.2.

Generally, the optoacoustic process can be separated into two fairly independent stages: the formation of the initial pressure distribution—the optical branch—and the transformation of the traveling acoustic wave on its way to the detector—the acoustic branch. When optoacoustics is applied to calculate optical properties from an optoacoustic transient, the optical branch is the main limiting factor for these attempts [95]. As pointed out before, for measurements on human skin, the straightforward fitting approach can only be followed for the special case of optical attenuation in the ultraviolet wavelength range: The high absorption coefficients here allow the neglect of scattering so that only one unknown variable—the absorption coefficient—is left in the theoretical description of the optoacoustic wave. Moreover, the absorption coefficient can be considered constant within the resolution limits (approximately 20 µm) of the used setup. As can be seen from Figure 17, penetration depths are below 60 µm for the forearm and high optical contrast from surface to inner tissue would be needed to make differences in $\mu_{a}$ resolvable at this scale.

More challenging cases are found when moving to the visible or infrared part of the spectrum. Optical radiation penetrates much deeper into the tissue as the absorption coefficient is 1–2 orders of magnitude lower than in the ultraviolet range. Consequently, the scattering coefficient $\mu_{s}$ is on the same order of magnitude than $\mu_{a}$ or scattering even dominates over absorption and in any case, the optoacoustic problem does not have a unique solution anymore. This issue may be addressed by introducing *a priori* knowledge, e.g., about the scattering coefficient $\mu_{s}\left(z\right)$, which could be achieved by complementary measurements by OCT, for example (see Section 4.4.1). The intensity of the radiation within the medium may be represented as the sum of the intensities of a ballistic term, describing photons which have not yet been scattered, and the diffuse scattered light field. However, if scattering can be neglected, as in the case of ultraviolet photons in human skin, calculations can be done based on the first term only. The ballistic component decreases exponentially with increasing depth (Lambert-Beer-law). If scattering cannot be neglected, the solution of the second term may be found by solving the diffusion equation obtained from the familiar radiative transfer equation considered in the diffusion approximation subject to the conditions that $\mu_{a}\ll \mu_{s}^{\prime }$ and that the distribution of the sources is isotropic. However, the diffusion approximation can only be used to calculate the spatial distribution of the light intensity in a turbid medium at depths far from the surface at which the collimated radiation incident on the medium has been transformed into diffuse radiation. Numerical approaches such as Monte Carlo simulations are needed to calculate the light distribution in the sub-surface region. Still, the optical characteristics of an isotropic turbid medium, namely the absorption coefficient and the reduced scattering coefficient, may be calculated from the profile of the leading edge of the optoacoustic pressure signal in the diffusion approximation recorded with a high temporal resolution [108]. However, when depth-dependent attenuation coefficients need to be considered, analytical solutions are no longer available and even numerical approaches become scarce. So far, at least *a priori* knowledge about the expected depth dependence of the optical properties (e.g., several layers with predefined thickness) is needed. Intelligent guessing approaches and more computing power may help to better approximate depth-dependent optical properties in the future.

### 3.2. Calculation of Optical Properties from Optoacoustic Measurements—Inverse Solution

**Figure 19:**
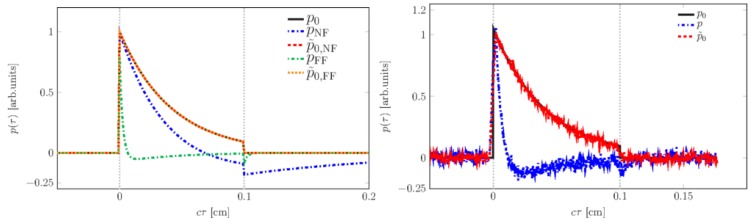
Inverse reconstruction of the initial stress profile (${\tilde{p}}_{0,NF}$,${\tilde{p}}_{0.FF}$) from calculated optoacoustic signals ($p_{NF}$,$p_{FF}$) in the near and far field, respectively, matching perfectly the true initial stress profile $p_{0}$ (**left**). The **right** panel shows that the reconstruction also works convincingly with signals including a realistic noise component (Gaussian white noise, S/N:5) (reproduced with permission from [107]).

The ideal approach towards measurement of optical properties from optoacoustic data would be the complete inverse solution of the optoacoustic problem. This would allow direct calculation of the underlying depth-dependent optical properties from a given optoacoustic signal. As pointed out already, the light distribution and consequently the formation of the initial pressure distribution cannot always be described analytically and besides, for non-negligible, depth-dependent absorption and scattering coefficients $\mu_{a}\left(z\right)$ and $\mu_{s}\left(z\right)$ there may exist multiple solutions. So, inversion is difficult at this point. However, it is generally useful to find an inverse solution for the transformation of the acoustic traveling wave on its way to the detector yielding the initial pressure distribution as this marks a central endpoint which may then be further analyzed by another inversion scheme or by fitting with numerical or analytical data. This also serves as a basis for optimal processing of optoacoustic data for imaging applications. To do so, it is useful to stay with an on-axis setting for the positioning of the detector with respect to the optoacoustic source volume. This does not imply complications of the experimental setup. In fact, our current setup for clinical use (see below) already uses on-axis or close to on-axis illumination. In [107], we present an effectively one-dimensional approach for an approximate but highly efficient inversion of observed optoacoustic signals to initial stress profiles for the full 3D optoacoustic problem. Equation (1) can be rewritten in the form

(4) $$p_{D}\left(\tau \right)=\alpha \left(p_{0}\left(\tau \right)-\int_{-\infty }^{\tau }K\left(t,\tau \right)p_{0}\left(t\right)dt\right)$$

showing that the initial pressure distribution $p_{0}$ experiences a diffraction transformation as described by the second term taking the form of a Volterra operator. It governs the propagation of acoustic stress waves in the optoacoustic on-axis setting with a Gaussian irradiation source profile via a convolution-type Volterra kernel K(t,τ)=K(τ−t). Based on the 1D Volterra integral equation, it was possible to reconstruct the initial stress profiles from synthetic signals with an algorithm terminating in time O(N). Figure 19 shows that this approach reconstructs the initial pressure distribution with great accuracy for synthetic FF and NF data.

**Figure 20:**
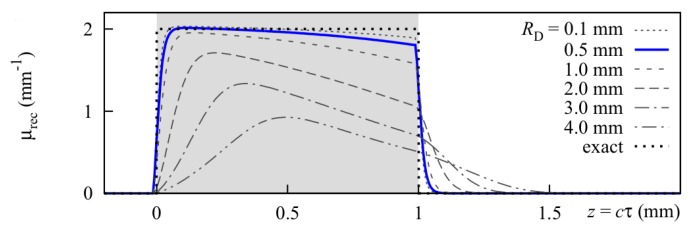
Absorption coefficient profile $\mu_{a}\left(s\right)$ as reconstructed from the initial pressure profile obtained by the inverse solution in terms of the optoacoustic Volterra integral equation. The detector radius $R_{D}$ is crucial for the accuracy of the reconstruction. Realistic dimensions as found in our optoacoustic setup for clinical application are marked in blue and still show very good agreement to the exact (constant) result (reproduced with permission from [107]).

Experimental conditions in this case also allow for inversion of the optical branch. Therefore, the depth-dependent absorption coefficient $\mu_{a}\left(z\right)$ can be reconstructed from the initial pressure profile retrieved by inversion of the acoustic part of the optoacoustic problem (Figure 20).

Therefore, we succeeded in implementing a fast inversion scheme fitting our optoacoustic experimental setup including typical S/N. Assuming negligible scattering, even absorption coefficients could be derived. As pointed out before, complementary estimation of the scattering coefficient $\mu_{s}$ via OCT or other methods could remove the restriction of this approach concerning the optical properties and enable transfer to reconstruct any set of optical properties.

### 3.3. Depth Profiling in Dermatology via Optoacoustic Measurements

It needs to be pointed out though that for many applications, not the absolute optical properties need to be deduced from a sample, but rather significant changes in the optical properties caused by the morphologic structure of a tissue. For example, if pigmented and non-pigmented tissue can be discriminated by an optoacoustic measurement, it is possible to measure the thickness of a pigmented skin lesion non-invasively. Such measurements provide highly interesting extra information for skin cancer screenings, for example. Dermatologists usually assess suspicious nevi by epiluminescence microscopy (dermoscopy). Based on the outcome of this visual inspection, they decide for a biopsy to be taken to confirm or refute melanoma skin cancer. The thickness or invasiveness of the lesion is the primary factor determining the risk of metastasis—the main cause of death from this tumor type—and yields the tumor stage [109]. It is measured using histopathology, so results are usually available within a few days. If this thickness could be determined pre-surgically, the psychological burden on the patient could be minimized and safety margins of the lesion may already be applied at the first excision. It needs to be pointed out in this respect that even with an experienced dermatologist, only one suspicious lesion in 20 are confirmed as melanoma skin cancer. If lesions thinner than 1 mm are excised properly, the risk of metastasis is very low.

**Figure 21:**
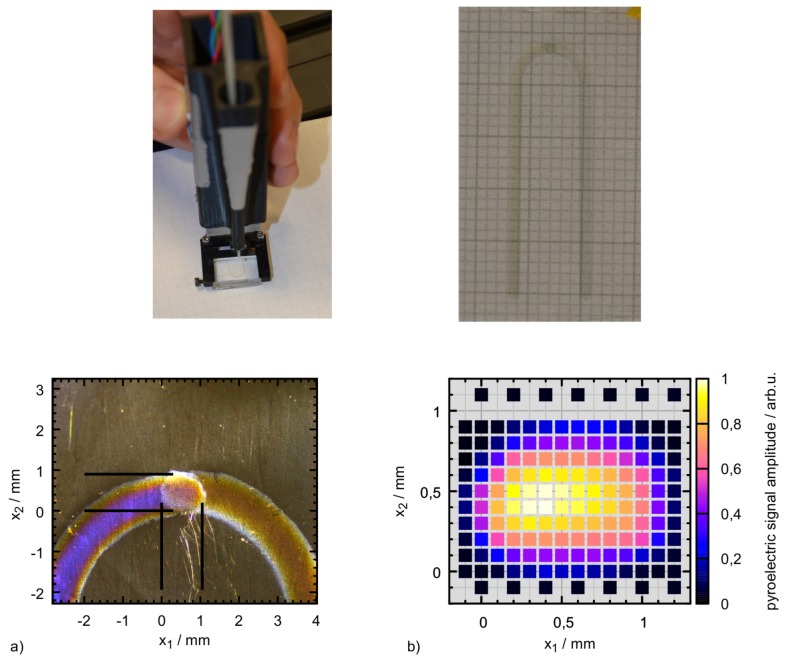
The **top left** panel shows a photograph of the optoacoustic hand-held device developed for clinical applications. An enlarged view of the sensitive element pre-mounting is shown in the **top right** panel. It lies on mm paper to get a better impression of size and transparency. The **bottom** row shows an enlarged view of the electrodes at top and bottom sides of the sensor film forming the active area by their overlap (**left**). The active area is confirmed by measurement of the pyroelectric effect induced by direct raster illumination of the electrodes on film through 150 × 150 µm apertures (**right**) (reproduced with permission from [111]).

For this application in clinical dermatology, we developed a hand-held device including a transparent detector to facilitate targeting of optoacoustic measurements on the nevus or lesion. The standard optoacoustic detector is a metal-coated piezoelectric film [93]. The sensor element we implemented consists of a polyvinylidene fluoride (PVDF) film which is transparent by itself (>85% transmission in the VIS) and indium tin oxide (ITO) electrodes sputtered on its surface. A similar concept was proposed by Martin Frenz’ group [110]. In comparison, our sensor setup was optimized for better spatial resolution using a thinner, 9 µm, PVDF film, the active area of the sensor was reduced significantly to 1 mm diameter to facilitate accomplishment of NF conditions for the measurements and we introduced a sandwich design for the film to increase robustness in later clinical operation. The sensor is mounted to a 3D printed support providing connection to the customized electronics and guiding the illumination fiber. Figure 21 illustrates the hand-held detector device.

A frequency-doubled pulsed Nd:YAG laser is used in this setup. Excitation at 532 nm was chosen attempting a good compromise between favorable high penetration depths in human skin and good contrast by strong absorption.

**Figure 22:**
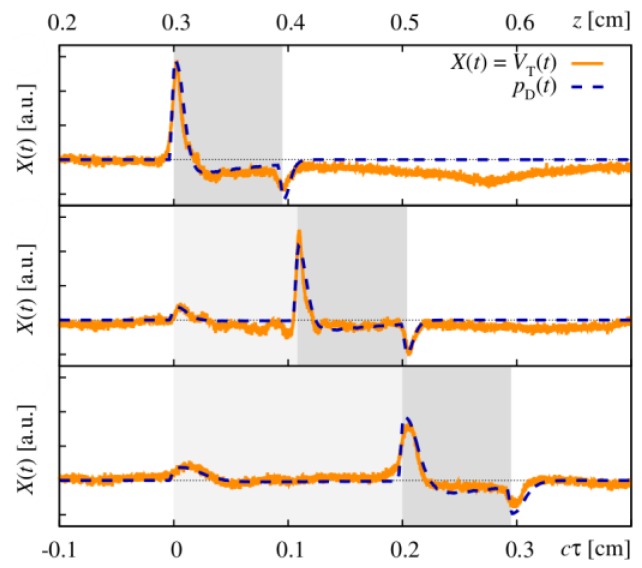
Experimental (yellow curve) and numerically calculated (dashed line) optoacoustic signals of poly(vinyl alcohol) (PVA) hydrogel layers with different absorption properties (different shades of gray) realized by melanin staining and measured with a detector in the far field. Melanin concentration in the different layers was chosen to match physiologic conditions (reproduced with permission from [106]).

The optoacoustic setup was employed for a pilot study at the Clinic for Dermatology and Venereology, University Medical Center Rostock in cooperation with Prof Dr Steffen Emmert. Optoacoustic measurements were carried out on pigmented nevi prior to their excision. The depth of the pigmented nevus was determined by histopathologic analysis, the gold standard for depth measurement in current dermatology. Analysis of optoacoustic data and possible verification of results against the histopathologic data is still ongoing. However, we could already show in both numerical simulation and optoacoustic FF measurements on in vitro tissue phantoms that the border of layers of mm thickness and with different absorption properties can be found clearly in the optoacoustic signal (Figure 22) [106].

As can be seen, layer boundaries marked by a significant change in absorption properties cause peaks or dips in the optoacoustic signal depending on whether the absorption coefficient is increased or lowered in the following layer. The absorption coefficient for the layer representing the pigmented nevus were chosen to be ten times higher than that of the layer representing the surrounding normal skin. As far as absorption is concerned, this can be considered a realistic scenario [112]. So far, the PVA hydrogel model has not included scattering and, even more importantly, the layers have been homogeneous. As skin consists of different skin layers with different optical properties, choosing a layered structure for in vitro or in silico modeling is the standard approach [113,114], still the model simplifies strongly the underlying inhomogeneous cell structure of the tissue.

Therefore, summarizing, we have already shown the potential of optoacoustics as a quantitative measurement tool for in vivo applications in dermatology from preparing the numerical tools for presurgical depth determination of melanoma thickness [106] to the determination of optical properties of human skin in scenarios that allow for significant simplification of the theoretical description of optoacoustic signals [103,104,105] and in the more general case suggesting solutions for the inverse problem [107].

Combination with other modalities provides significant mutual benefit as will be outlined in the following chapter.

## 4. Multimodal Applications—Consecutive Information Flow and Parallel Multi-Source Investigations

### 4.1. Tissue Phantoms for Multimodal Verification

Before application of optical spectroscopy in situ, it is imperative to validate the method using defined, well-characterized reference samples. These reference samples are to be tailored to the requirements of the application and to the characteristics of the methods. Multimodal approaches implicate even higher demands towards reference samples as these need to comply with the requirements of each of the modalities involved.

Raman spectroscopy and optoacoustics is a multimodal combination that is particularly interesting for quantitative studies on biomolecules in vivo. Optical properties of biological samples can be measured non-invasively and in situ by optoacoustics. Based on this data, the intensity of in situ Raman spectra can be corrected for losses due to signal reabsorption and scattering. If this attenuation is known, the concentrations of the Raman-active molecules can be calculated.

**Figure 23:**
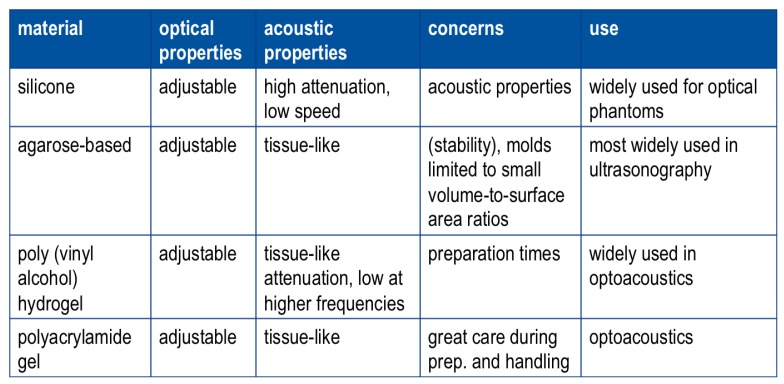
Comparison of possible materials for soft-tissue-like reference samples to be used for both optical and acoustic methods.

Liquids such as ink, intralipid, or others are often used as most simple reference samples. However, they only perform well, when the sample is to represent only one homogeneous layer. Therefore, they constitute a good starting point, failing however at tasks where some of the complexity of real tissue is to be represented, i.e., at more complex layered or inhomogeneous structures. Solid samples can be prepared with inhomogeneous, tissue-like optical properties, but often fail at providing appropriate acoustic properties as demanded by optoacoustics. Hydrogels have tissue-like acoustic properties due to their high water content and, in comparison to liquid samples, the advantage of being stackable to produce layered structures. In contrast to liquids, hydrogels allow abrupt changes of optical properties from layer to layer only softened by diffusion of solved ingredients. Many different materials are available and in use for tissue phantoms for optical spectroscopy [115]. Given the additional requirements of acoustic techniques such as ultrasound imaging and photoacoustics, the list reduces to agar, silicone, poly(vinyl alcohol) gel (PVA) and polyacrylamide gel (PAA) [116]. Figure 23 summarizes their applicability as soft tissue phantom materials.

PVA is one of the most promising materials for combination of acoustic and optical techniques. Even though preparation is more time-consuming than with other materials, it provides excellent acoustic properties and good longevity, and its optical properties can be well adjusted. Water-soluble absorbers are best suited to adjust the hydrogel’s absorption properties, and for approaches that are insensitive to microinhomogeneities, a large variety of even insoluble but biologically relevant molecules such as melanin may be introduced by dispersion. Scattering coefficients can be adjusted by adding intralipid or microspheres. Besides, the turbidity of PVA hydrogel itself is dependent on the number of freeze-thaw cycles during its preparation [117] and may also be used to adjust scattering properties.

As indicated in Section 2.3, the quantification of carotenoids in situ is interesting from both a biomedical and an environmental point of view. Therefore, we developed a method to introduce the water-insoluble carotenoids into PVA hydrogels for use as reference samples for these studies [118]. We modified the well-known protocol by Kharine et al. described in [117] replacing dimethyl sulfoxide (DMSO) with ethanol as anti-freezing and pro-polymerization agent. This improves handling and reproducibility of the reference samples based on these hydrogels as it allows production of hydrogels without the need for a final rinsing procedure in order to remove the DMSO. During rinsing, dye molecules may be washed out with the DMSO or swelling of the hydrogel in the water bath may occur leading to unwanted increased turbidity of the phantom. The water:ethanol ratio was varied for experiments. A clear hydrogel is produced with ca. 25–40% *v/v* of ethanol in aqueous solution. In [106], we successfully made use of such clear absorbing hydrogels, which were partly stained by different concentrations of melanin, to verify numerical simulations by comparison to measured optoacoustic signals, so these PVA hydrogels perform well as a reference for optoacoustic studies. For evaluation of the hydrogels for Raman spectroscopic studies, we formulated samples with 12% *v/v* of ethanol, so they were slightly turbid. Carotenoid-stained hydrogels were produced by (partly) replacing the pure ethanol by a solution of β-carotene in ethanol, or by introducing an emulsion of β-carotene during hydrogel preparation. Only comparably weak Raman signals could be collected from hydrogels stained by ethanol solution of β-carotene because of the low concentration caused by solubility limits and oxidation during the hydrogel production process. Introduction of β-carotene in emulsion allows a much more intense stain, as the solubility of β-carotene in lipids (>1 g/L) [119] is much higher than in ethanol (30 mg/L) [78]. The Raman spectral characteristics of β-carotene in PVA hydrogel stained by emulsion of β-carotene or β-carotene dissolved in ethanol are very similar to the characteristics of the diluted emulsion or solution, respectively (see Section 2.3.1 and Figure 11 and Figure 12).

**Figure 24:**
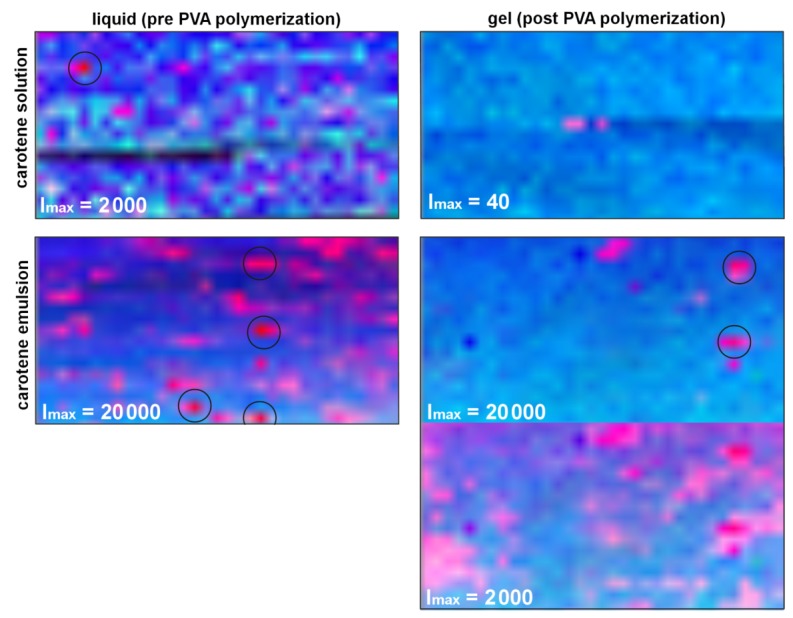
Raman images of liquid (**left**) and polymerized (**right**) PVA hydrogels that were stained with β-carotene solved in ethanol (**top**) or β-carotene in emulsion (**bottom**). Water is color-coded in blue and PVA in green yielding turquoise for the hydrogel matrix. The distribution of β-carotene is shown in red. As color saturation encodes signal intensity, carotene distribution appears as different shades of pink in dependence of the β-carotene concentration. Please note that full color saturation refers to different maximal carotenoid signal intensities I${}_{max}$ (a.u.) in the different images. Circles mark carotenoid agglomerations where exceptionally high β-carotene concentration and very weak water and PVA concentrations are found. The double image at the bottom right shows the same image (polymerized PVA hydrogel stained with β-carotene in emulsion) at two different carotenoid color scales to illustrate the large local variability of β-carotene concentration in the hydrogel. Each image shows a 100 µm wide sub-surface area of the gels (reproduced with permission from [76]).

Beyond the spectral characteristics, we also studied the spatial distribution of the carotenoid within the hydrogel. The stain of the rigid hydrogel appeared to be homogeneous to the naked eye, so it can probably be declared homogeneous within the resolution of fiber-based Raman or optoacoustic measurements as intended for use as a skin tissue or plant phantom. However, the carotenoid distribution or the hydrogel matrix may not be claimed homogeneous with respect to confocal Raman microspectroscopy given its much higher spatial resolution. Therefore, we analyzed the β-carotene distribution in hydrogels stained with β-carotene solution and emulsion prior and post polymerization by confocal Raman microspectroscopy at 532 nm excitation (see Figure 24).

This investigation also gave an impression of the molecular environment of the carotenoids in the hydrogel. As pointed out before, this is crucial for their optical properties and possible aggregation may have a strong impact on carotenoid absorption and thus Raman resonance. In the phantom, the carotenoids are exposed to both water and ethanol as well as the PVA matrix. For the phantoms stained with carotenoid emulsion, the lipid environment is expected to play an additional important role. While water and carotenoids can be identified unambiguously by their Raman signal, PVA and ethanol share most of their Raman lines and are thus difficult to discriminate. PVA can be identified by an additional albeit weak line at a Raman shift of 926 cm${}^{-1}$ which is not present in the ethanol signal. Ethanol on the other hand does not show unique lines compared to PVA, so it was not possible to determine and analyze its distribution. As expected, water was evenly distributed in the hydrogels and the PVA polymer matrix showed a consistent micropattern in all hydrogel samples whether stained or not. Here and there, the polymer matrix contains water cavities, a few µm wide. Close to the hydrogel surface, i.e., within the first few micrometers, the PVA signal intensity is generally slightly increased. This is probably due to a denser polymer network at the surface. Figure 24 also depicts an exemplary distribution of β-carotene as found in the hydrogels showing that despite an overall homogeneous yellow-orange appearance of the hydrogels at the macroscopic scale, the β-carotene distribution is inhomogeneous and sometimes clearly structured at the microscale. This limits usability of this reference sample for confocal imaging if homogeneous conditions are to be mimicked. In hydrogel stained with β-carotene emulsion, the Raman fingerprint of β-carotene is present in every pixel be it before or after polymerization. After polymerization (induced by one freeze-thaw cycle), the Raman spectral intensity is considerably reduced in both cases. Absorption spectra indicate a carotenoid loss in the order of 30% probably due to oxidation.

In summary, here, we report the first successful attempt at incorporation of β-carotene in PVA hydrogel. The presented procedure can be transferred to other carotenoids easily. So far, only very few reports on microencapsulation of carotenoids can be found in the literature [120,121,122]. All of them are very recent and focus on nutrition and bioaccessibility of the carotenoids, so neither do they study the optical properties of carotenoids in the hydrogel nor are the chosen hydrogel materials compatible with e.g., optoacoustics. PVA hydrogels may well be used as reference samples for ’macroscopic’ Raman spectroscopy and optoacoustics, but are not suitable for confocal microscopy or other high spatial resolution modalities as the microinhomogeneity is too strong. In any case, care needs to be taken to adjust the optical properties as given by the target tissue.

### 4.2. Correction of Raman Spectra via Absorption Spectroscopy and Optoacoustics

As already reported (in the previous Section 3 and Section 4.1), optoacoustics can measure optical properties of tissue. Obtaining this information provides the basis for correction of Raman spectra for their spectral attenuation during propagation through the sample. The next step towards quantification of molecules via their Raman signals is to set up a model to calculate spectral signal attenuation. In the case of human skin, the light propagates through multiple layers with different compositions of hemoglobin, melanin, β-carotene and other chromophores [123]. Besides, cells, granules, and fibrous structures cause scattering. Thus, the Raman spectrum will not only be attenuated, but also spectrally deformed. One way to determine Raman signal attenuation is a Monte Carlo-based simulation of the photon trajectories. Monte Carlo modeling is widely used to simulate light transport in tissue including Raman scattering [59].

Building on the highly popular Monte Carlo package developed by Wang et al. [124], we implemented two different approaches for simulation of Raman scattering and evaluated their performance against each other to identify optimal parameter regimes for operation [125]. The MC methods were realized in graphics processor unit (GPU) environment to minimize calculation time [126]. Extension to our specific task included implementation of light source and detector geometry, extension to generation of inelastically scattered photons, and most importantly a spectral input format to accommodate spectrally dependent optical properties.

The ’two-step approach’ was inspired by work of Reble et al. [127]. In a first step, the distribution of the absorbed excitation light is calculated. In the second step, Raman photons are launched from each grid element of the sample according to the excitation photon fluence, which is derived from step one, and the Raman scattering coefficient. To improve the statistics, our algorithm allocates any number of photon packages to the grid, scaling up but keeping the distribution of the excitation fluence. The Raman photons of step two are launched with isotropic direction and randomly chosen wavelength. The probability density function for the wavelength selection is given by a measured Raman spectrum.

**Figure 25:**
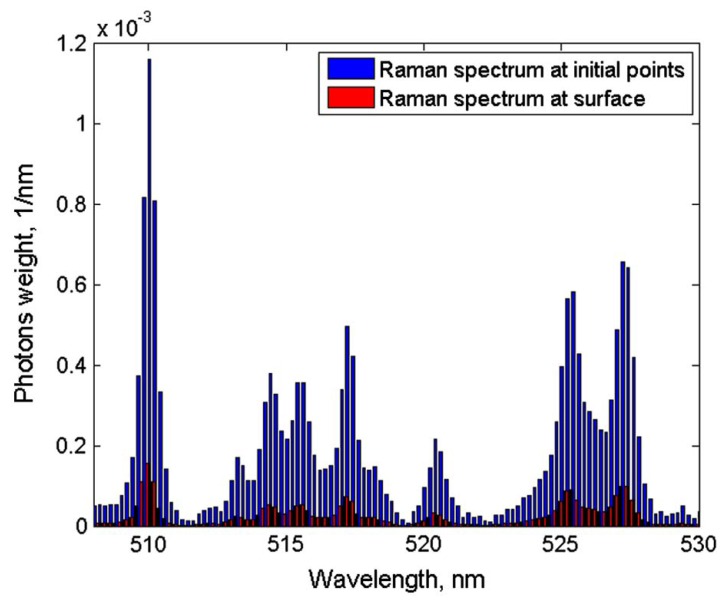
Simulation of Raman spectra by the single-pass MC method: input Raman spectrum of β-carotene dissolved in ethanol (blue), and the numerically obtained Raman spectrum at the surface (red) (reproduced with permission from [125]).

In the ’direct approach’ Raman photons are generated randomly during propagation of the exciting photon package. Isotropic molecular scattering, Raman shift selection, and propagation at shifted wavelength and according optical properties are applied as in the two-step approach. In contrast to the two-step approach, the Raman scattering probability is assumed to be orders of magnitude larger than in reality to generate reasonable statistics in this single-pass approach, which can be justified according to Everall et al. [128]. Figure 25 compares the Raman spectrum of β-carotene measured at the surface of a cuvette at excitation with 488 nm radiation to the input Raman spectrum indicating the probability density distribution.

Results from both approaches are in overall good agreement and both approaches work with acceptable photon statistics. The two-step approach has the advantage that a good statistic representation of the stochastic events can be achieved even for realistic Raman scattering probabilities. The single-pass approach, on the other hand, appears to model the physics of photon propagation more truly.

Both approaches can calculate the deformation of the Raman spectrum in a semi-infinite multilayer model. In the future, the idea is to reckon back an intrinsic Raman spectrum from the observed Raman spectrum [129] and to estimate the concentration of any analyte inside the examined sample in this way.

**Figure 26:**
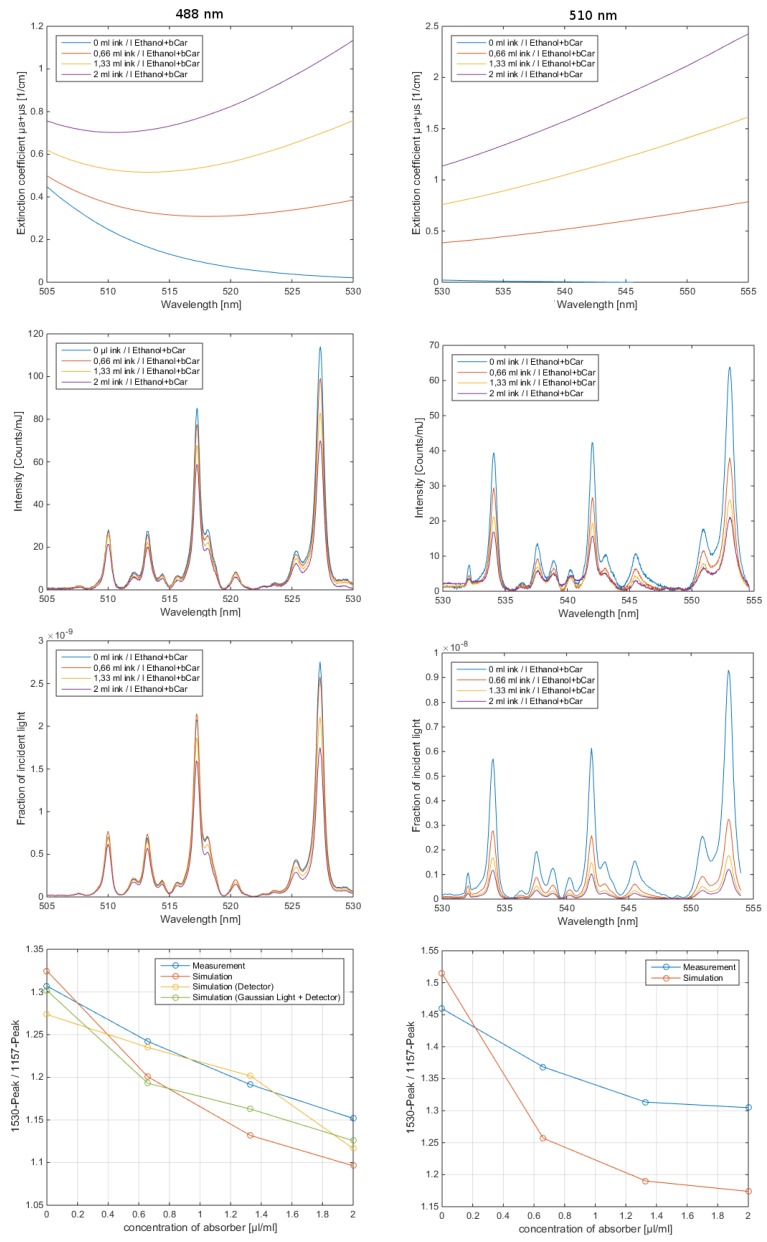
Correction of β-carotene Raman spectra for attenuation during propagation through an ink stained liquid sample at 488 nm (**left**) and 510 nm (**right**) excitation. The **top** row shows the absorption spectrum of the sample in the spectral range of the calculated β-carotene Raman spectrum. The **second** row shows experimental data to be compared to the calculated spectra in the **third** row. The bottom row shows the peak ratio of the two strongest β-carotene Raman peaks for experimental and simulated data. For 488 nm excitation, the effect of inclusion of a finite size detector and illumination by a Gaussian beam is shown as well.

Beyond the self-absorption effects already seen in Figure 25, we attempted to validate the two-step algorithm by comparing simulated output spectra and resonance Raman measurements for homogeneous samples with different absorption spectra (see Figure 26).

Absorption spectroscopy was used to determine the extinction coefficients of β-carotene solution that was additionally stained by addition of ink at different concentrations and Raman spectra were excited at different wavelength. Numerical simulation via the two-step MC approach (Figure 26, 3rd row) yielded output spectra that are qualitatively already in good agreement with the experimentally measured spectra (Figure 26, 2nd row).

However, further development in the numerical model is still needed as significant details are not predicted with high enough accuracy. This is best seen when looking at the peak ratios of the strongest Raman peaks (Figure 26, bottom row). Considerable differences between simulated and experimental results are found in the development of the peak ratio of the carbon double and single bond vibrations. The effects of more realistic implementations of experimental conditions are shown exemplary for 488 nm excitation. These variations and discrepancies may not appear significant at first sight; however, correct reproduction of the peak ratios by the numerical model is prerequisite to become able to quantify molecules by their intrinsic Raman spectrum in the presence of other Raman-active molecules.

Our extension of the numerical MC model to comprise a full spectral representation of the Raman signal as described in this section is a significant advance compared to the previous work of other groups. So far, quantification was based on analysis of single Raman lines [127] and our results concerning e.g., the reproduction of the main peak ratios indicate that the ability for spectral analysis is important for both further improving the model as well as the prospect of discriminating different molecular contributions to coinciding Raman peaks by aid of full spectral information.

### 4.3. Simulation of Vitamin D${}_{3}$ Photosynthesis

Photochemical activity in plant or animal tissue depends on the availability of light energy and photoconvertible molecules. Both these parameters vary substantially within the tissue volume and are difficult to determine in the individual tissue. Action spectroscopy investigates the spectral efficiency for inducing a specific photochemical or photophysical effect. In the life sciences, the effect is often a photobiological physiological endpoint such as photosynthesis rate, sunburn (erythema) or vitamin D${}_{3}$ production. Of course, it is generally desirable to study such photobiologic processes directly (as shown in Figure 13 with respect to the violaxanthin cycle). However, it may also be highly instructive to create computer models of tissue to study photochemical processes and their parameters. In this section, we show how optoacoustically measured data on UV absorption in tissue is integrated in a model for vitamin D production in human skin. This model may in the future be refined and enhanced by introducing e.g., data on the individual distribution of previtamin D derived from Raman spectroscopic data.

Vitamin D is important for bone health and our immune system and low levels of vitamin D have been implicated in a wide variety of health issues including cancer, diabetes, and cardiovascular disease [130,131,132]. Most of the vitamin D circulating or stored in our bodies is produced by UVB irradiation of our skin. Inadequate vitamin D levels are prevalent especially in Europe [133] mainly due to behavioral effects, age, and skin phototype [134]. As ultraviolet radiation also causes skin damage leading to skin cancer, erythema, and photoaging, sunbathing may not be considered first choice in reaching and maintaining optimal vitamin D status. The dilemma between positive health effects and risks of ultraviolet exposure is one of the key questions in current photodermatology including an ongoing controversy about the implications and validity of (standard) action spectra of UV effects [135,136,137,138]. Basically, both vitamin D production and negative UV effects are efficient in a very similar spectral range. Effectiveness in both cases is strongly individual depending on the skin phototype and/or ethnic origin and depends on the spectral quality of the UV exposure. Advice for the personal, individual balance between risk and benefit heavily build on these dependencies based on measures such as the skin phototype and the UV index. However, the reference action spectra generally do not represent the inter-individual differences. Therefore, efforts are made to model vitamin D photoproduction accounting for these different parameters and compare results to the standard action spectrum. One challenge here is the lack of in vivo data for almost all parameters, e.g., provitamin D amount, distribution, and supply kinetics in skin. Fortunately, our optoacoustic measurement of optical properties of human skin (see Section 3.1) fills one of these gaps providing in vivo attenuation data for human skin of the Caucasian phototypes I-IV at three different skin sites.

**Figure 27:**
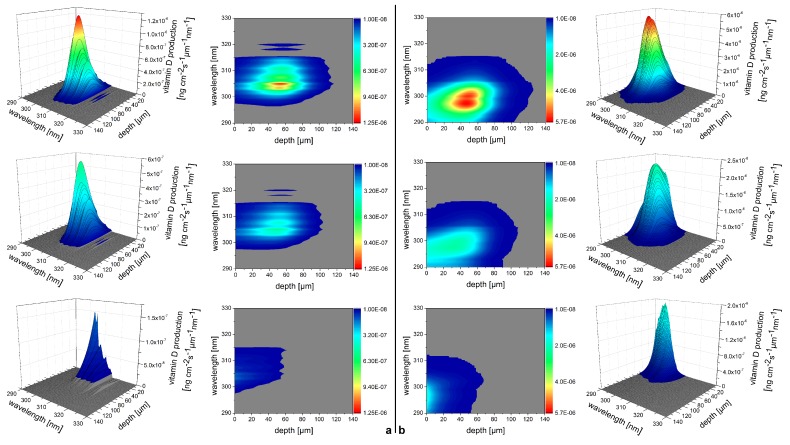
Modeled previtamin D photoproduction. Production by solar irradiation is shown in 3D and as contour plots at the **left** side (**a**), production from a TL12 “fluorescent sun” lamp is shown on the **right** side (**b**). Different skin sites are shown: inner side of forearm (**top** row), outer side of forearm (**middle**), ball of the thumb (**bottom** row). The color-coding scale is different for TL-12 and solar irradiation but consistent within each group (reproduced with permission from [139]).

Building on this data, we set up a fairly simple model for previtamin D photoproduction in human skin [139]. We calculated the spectral irradiance as attenuated by the optical properties of the skin as a function of sub-surface depth assuming exponential attenuation by absorption only. The light distribution within the skin is then weighted spectrally with an in vitro action spectrum for previtamin D production [140,141] and weighted spatially with an ex vivo concentration profile of the educt provitamin D [142] to calculate the total amount of previtamin D produced in the skin for a given scenario of skin site and irradiation source. Figure 27 shows spectral depth profiles for previtamin D photoproduction for two irradiation sources and three different skin sites.

**Figure 28:**
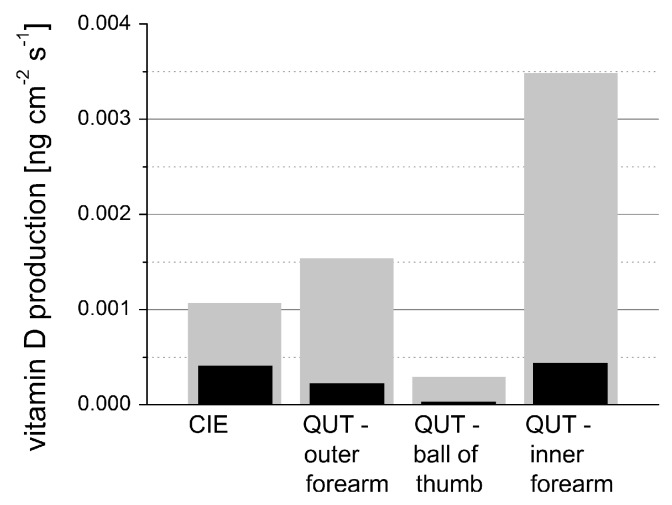
Total previtamin D production from solar (black) vs. artificial (gray) irradiation as predicted by our model (QUT) for different skin sites and assuming a step function (**left** panel) or a smooth (**right** panel) provitamin D concentration profile in the skin. Results from the CIE standard spectrum where skin properties are implicit are given for comparison (reproduced with permission from [139]).

Both the spectral composition of the irradiation source and the exposed skin site has considerable effect on the amount and localization of previtamin D photoproduction. The calculated contributions of the skin layers to overall previtamin D production qualitatively agrees with published data, i.e., most previtamin D is found in the *stratum spinosum* and *stratum basale*, the least in the dermis [142]. The total amount of produced previtamin D as calculated from our model and from application of the CIE standard action spectrum [143] is compared in Figure 28.

Predicted previtamin D production for weakly pigmented skin (inner forearm) exposed to solar radiation (black) agrees well to that calculated with the CIE action spectrum. However, other skin sites with different optical properties are predicted to produce considerably less previtamin D. The outer side of the forearm which—in daily life—is exposed to vitamin D effective UV radiation much longer than the inner side produces less than half the amount predicted by the CIE spectrum under the same irradiation conditions. So far, we have not undertaken attempts to verify our results by in vivo measurements of previtamin D production. Still, the results underscore the importance of individual factors for the estimation of vitamin D production which is especially important with respect to generalized health advice based on the standard action spectrum.

Our model for the first time allows in-depth assessment of the influence of individual aspects such as skin phototype or skin site on vitamin D production. It took some years until only recently, van Dijk et al. [144] showed the topicality of the discussion on the standard vitamin D action spectrum presenting a more elaborate model for prediction of vitamin D photoproduction in silico and comparison with results from different action spectra.

### 4.4. OCT for Multimodal Measurement and Imaging

As outlined throughout this work, multimodal applications benefit research either by applying information obtained by one modality to improve results obtained by another or by providing multi-source complementary information on the same subject. Another optical modality that can provide both benefits is OCT. In our work presented in the following subsections, it serves as a measurement instrument for determination of non-linear scattering coefficients and is applied to measure the depth of human skin nevi in vivo in a comparative study with high-frequency ultrasound. Moreover, we developed a multimodal setup allowing combination of in vivo imaging of skin morphology by OCT with the diagnostic potential of Raman spectroscopy.

#### 4.4.1. Prediction of Scattering Coefficient via OCT

Recently, attempts have been made to extend the field of application of OCT from a pure imaging to a measurement modality by extracting optical properties from the recorded image. Both analytical and numerical models have been applied, but many aspects have not yet been satisfactorily solved.

In low- or non-absorbing media, the intensity decay of the OCT signal may be analyzed to derive the scattering coefficient of a sample assuming an exponential decay. This would be straightforward for ballistic photons. However, single and multiple scattered photons contribute to the OCT signal posing the main challenge for conclusive and realistic simulation of OCT signal generation. Scattering events lead to a decrease in the degree of coherence of the OCT photons and consequently degrade the OCT signal. Multiple scattered photons cannot be neglected as this would lead to overestimation of the signal decay. One approach to account for the effect of multiple scattered photons and their differing contribution is categorization, e.g., in least-scattered photons (LSPs) and multiple-scattered photons (MSPs) [145,146]. LSPs are detected photons backscattered from a layer at the targeted depth *z*. This includes single-scattered photons and photons with few scattering events leading to differences between the optical pathlength traveled and the optical target depth of less than the coherence length. In contrast, MSPs fulfill the coherence condition for detection even though they have not been backscattered from the target depth *z* but above due to multiple wide-angle scattering events leading to a less straight optical path. Therefore, MSP are detected interferometrically but assigned to a scattering depth they have not reached. Consequently, MSP lead to a degradation of the detected OCT signal.

As a first step towards measurement of scattering coefficients from OCT data, we simulated an OCT A-scan based on the stochastic Monte Carlo method presented in [147] and take into account the geometrical constraints of our OCT detection system. We propose a new approach for the incorporation of multiple scattering events by means of a suitable heuristic weighting function. Our simulation code is based on Alerstams parallel GPU MC approach [126]. It efficiently calculates the position and direction of scattered photons at the surface of a non-absorbing semi-infinite medium. We calculate the simulated OCT signal by

(5) $$R_{sim}\left(z_{s}\right)=\sum_{n=1}^{n_{max}}\left[LSP\left(z,n\right)+MWP\left(z,n\right)\right]\times F_{W}\left(n\right)$$

counting photons belonging to the LSP or MSP group that were scattered up to *n* = 10 times and with a path length corresponding to the depth *z* and introducing the heuristic weighting function

(6) $$F_{W}\left(n\right)=\frac{1}{1+e^{a\times (n-b)}},$$

with *a* and *b* being fitting parameters. This weighting function reduces the contribution of MSPs with increasing number of scattering events. To determine the scattering coefficient $\mu_{s}^{sim}$ from the simulation data, $R_{sim}\left(z\right)$ is fitted with a function of the form

(7) $$R_{sim}=Ce^{\mu_{s}^{sim}\frac{z}{n_{m}}}$$

with C denoting an arbitrary scaling factor and $n_{m}$ the refractive index of the sample medium. Therefore, multiple scattering is taken into account in the simulation, but dependent scattering is not explicitly included within our physical model as described for example by Kalkman et al. [148]. The weighting function implicitly accounts for dependent scattering while reducing the numerical effort compared to more complex physical models based, for example, on an analytical extended Huygens–Fresnel approach [149].

A dilution series of milk in water was used as reference samples to establish the model. Scattering coefficients $\mu_{s}$ and anisotropy factor *g* were measured by a goniometer for samples containing up to 10% milk and $\mu_{s}$ was linearly extrapolated for less diluted samples. All simulations were based on this linear scattering coefficient $\mu_{s}^{lin}$, even though a non-linear (saturation) behavior is expected for high $\mu_{s}$.

**Figure 29:**
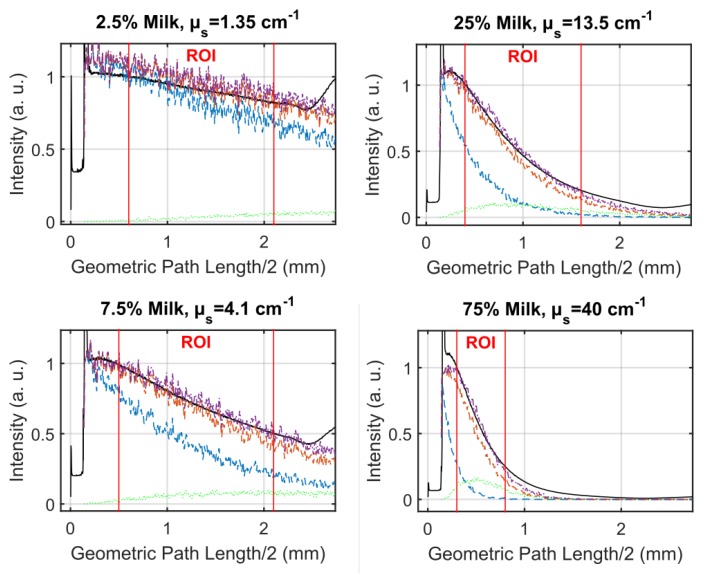
Contribution of different photon categories to the simulated OCT signal (purple) for different $\mu_{s}^{lin}$: MSP (green), LSP (red), ballistic (blue). The deconvolved OCT signal is shown in black and the region of interest (ROI) for calculation of the scattering coefficient of the sample assuming a pure exponential decay is marked by red vertical lines (reproduced with permission from [150]).

Figure 29 shows the contribution of least and MSPs to the simulated OCT signal depending on the milk concentration. As can be seen, the contribution of the MSPs to the signal increases for stronger scattering, and so does the discrepancy between measured signal and simulated signal if only ballistic photons are taken into account. Optimization of parameters *a* and *b* of the weighting function $F_{W}$ was guided by aiming at minimizing the difference between the scattering coefficient $\mu_{s}^{sim}$ derived from the simulated data and $\mu_{s}^{OCT}$ derived from the OCT measurement in the ROI. We found that parameter *b* becomes independent of *a* for large enough values of a (a≥5). For constant *a*, a linear dependence of parameter *b* on the linear scattering coefficient $\mu_{s}^{lin}$ is found for all concentrations.

**Figure 30:**
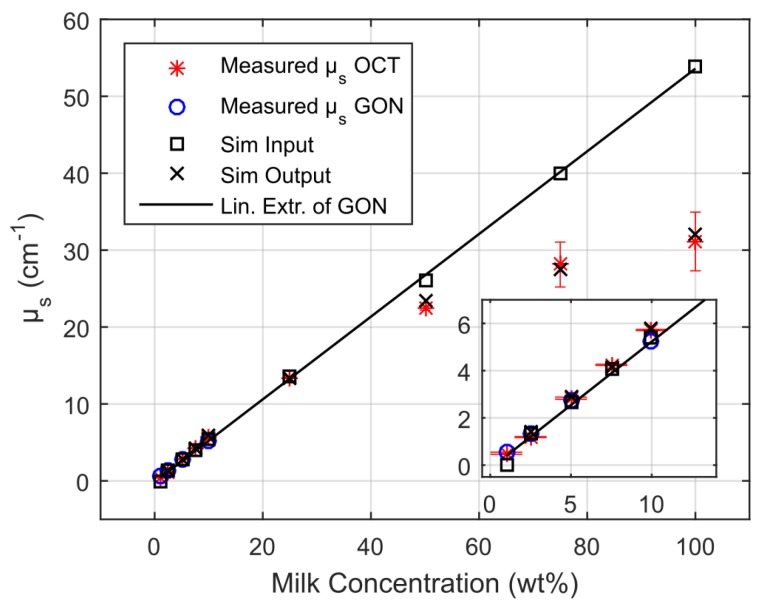
Comparison of $\mu_{s}$ obtained from goniometer (GON) measurements, simulation input data linearly extrapolated from these measurements (Sim input, $\mu_{s}^{lin}$), simulation results (Sim output, $\mu_{s}^{sim}$) and OCT measurements ($\mu_{s}^{OCT}$). The black line marks linear extrapolation from the goniometer measurements. Details of the data for low milk concentration are presented in the inset (reproduced with permission from [150]).

Figure 30 compares the scattering coefficients obtained by reference measurements with those derived from simulation data and OCT measurements. Remember that the simulation took linear scattering coefficients $\mu_{s}^{lin}$ as input parameter, which are derived from goniometer measurements and their linear extrapolation for higher concentrations and scattering coefficients, respectively. As can be seen, both the scattering coefficients $\mu_{s}^{OCT}$ derived from OCT measurements and $\mu_{s}^{sim}$ derived from the simulation show the expected non-linear saturation behavior in good agreement.

Therefore, our approach proved to be capable of predicting the non-linear concentration dependence of the scattering coefficient $\mu_{s}$ for different concentrations of a homogeneous sample with specified anisotropy *g* based on measurement of $\mu_{s}$ in the linear regime. A training set of experimental data is necessary to determine the parameters of the weighting function. For inhomogeneous samples or if absorption cannot be neglected, application of the approach must be carefully pondered. Estimations of average scattering coefficients may be feasible for weak absorption conditions. Still, such estimation of optical scattering coefficients by aid of OCT data can complement measurement of absorption coefficients by optoacoustics to quantitatively characterize light attenuation in tissue in vivo.

#### 4.4.2. Comparative Multimodal Studies of Tissue Thickness via OCT and High-Frequency Ultrasound

Measurement of distances such as depth or thickness by optical means is highly attractive for biomedical applications as already motivated in Section 3.3: Optoacoustics may support melanoma screening by presurgical measurement of the thickness of pigmented nevi. While in optoacoustics, contrast is generated by differences in absorption properties, OCT may be applied for this task based on refractive index changes reflecting tissue morphology. Combination of these two methods yields complementary information as OCT has better resolution and can better depict tissue morphology, which is diagnostically interesting, while optoacoustic measurements can gather data from deeper within the tissue. Moreover, depigmented nevi do not provide good contrast in optoacoustic images, for example. Nevus depigmentation may occur due to the immune system detecting the malignancy and partially attacking the cancer cells. Besides, optoacoustic measurements can support or verify depth assignment in OCT images as, in optoacoustics, depth is calculated from the run-time of the acoustic transient based on the sound velocity in tissue which is not as variable as the optical refractive index determining measured depth in OCT data.

As a first step towards this combination, we compared data from high-frequency (100 MHz) ultrasound and OCT obtained in a preclinical pilot study on 24 volunteers (f = 14, m = 10) in cooperation with the Clinic for Dermatology, Venereology, and Allergology, University Medical Center Göttingen and at the Clinic for Dermatology and Venereology, University Medical Center Rostock [151]. A total of 32 melanocytic skin lesions, suspicious for malignancy, were identified by visual inspection in this group. Suspicious lesions were imaged by OCT and high-frequency ultrasound (HFUS), OCT images were post-processed in a multi-step procedure to enhance contrast, and the thickness of the nevi were determined from both imaging modalities. In HFUS, ultrasound line scanners are implemented allowing 2D images (slices) only. Therefore, the operator had to scan for the thickest region of the nevus and take a picture. OCT produces 3D images containing the whole nevus, so the thickest part could be localized during post-processing. Results were compared to the thickness determined by the gold standard: a histopathological section from the excised nevus. It should be borne in mind that the whole process of histopathological preparation, i.e., excision, fixation, paraffin embedding, and slicing leads to some deformation of the tissue, so typically 10% error is to be assumed for thickness assessment even with this standard method.

**Figure 31:**
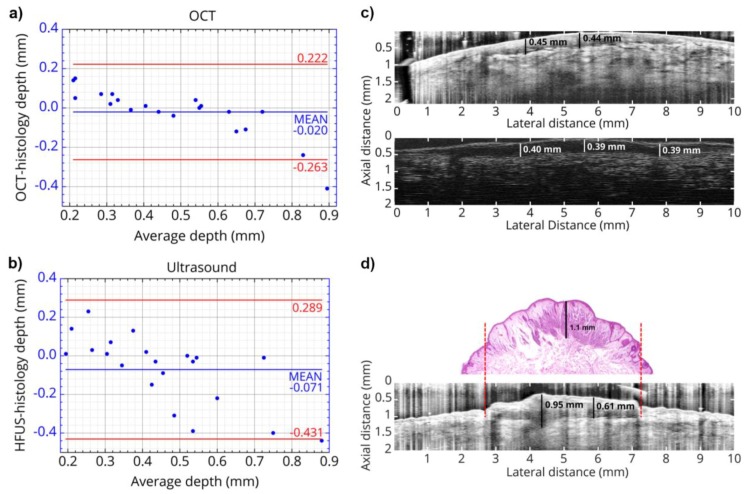
Determination of nevus thickness by OCT, HFUS, and histopathology. The Bland-Altman plots to the **left** describe the deviation of OCT (**a**) and HFUS (**b**) results from the nevus depth measured by histopathology. The **top right** pair of images (**c**) illustrate the appearance of a nevus viewed by OCT and HFUS, respectively. In the **bottom right** image (**d**), the stained histologicall slice is shown together with an OCT image of the same nevus with a thickness of approximately 1 mm visualizing also the deformations that can be induced by tissue preparation for histopathology. Corresponding regions for both modalities are marked by the red dashed lines (reproduced with permission from [151]).

A comparison of images and measurement results from the three modalities is summarized in Figure 31.

The thickness of thin nevi (<0.2 mm) is overestimated by both OCT and HFUS, whereas there is a trend towards underestimation with increasing thickness. On average, thickness is underestimated by both modalities with HFUS showing slightly larger deviation from the standard measurement than OCT. HFUS images have better contrast which helps identify the boundaries of skin lesion and, in contrast to OCT images, need no image post-processing. However, HFUS can only be applied consecutively with optical modalities impeding their combination. In contrast to this, combination of OCT with other optical methods is well possible as seen in the next section presenting our combination of OCT and Raman spectroscopy in a single device.

#### 4.4.3. Supporting Diagnostics by Combined Imaging and Spectroscopy

Beyond imaging, spectroscopic information can support diagnostics as well. Raman spectral analysis has made progress towards the discrimination of benign and cancerous tissue in skin cancer examination. According to a recent review be Schleusener et al. [152], Raman spectroscopy achieved similar results as trained dermatologists using dermoscopy. It should be kept in mind however that dermoscopy has a good sensitivity but a low specificity, e.g., sensitivity of 93% and specificity of 42% [153]. Therefore, improvement of Raman specificity for skin cancer diagnosis is in the focus of current research. Fiber probes are often used for clinical Raman spectroscopy as they can be designed in a very compact way ensuring good accessibility even at difficult skin sites such as interdigital folds but need to be carefully designed with respect to the application requirements, e.g., sampling depth. However, the scattering and fluorescence from the fiber materials may compromise tissue Raman signals and need to be handled with great care. In general, the low probability of Raman scattering often leads to high integration times and exposures. However, application of Raman spectroscopy in vivo must comply with safety standards concerning the maximal permissible exposure (MPE). To achieve good signal-to-noise ratio from tissue in vivo without exceeding the MPE is one of the core challenges of Raman spectroscopy in biomedical research. This applies even more so when compromises concerning the setup are to be accepted attempting to combine Raman spectroscopy with other modalities in a single device.

**Figure 32:**
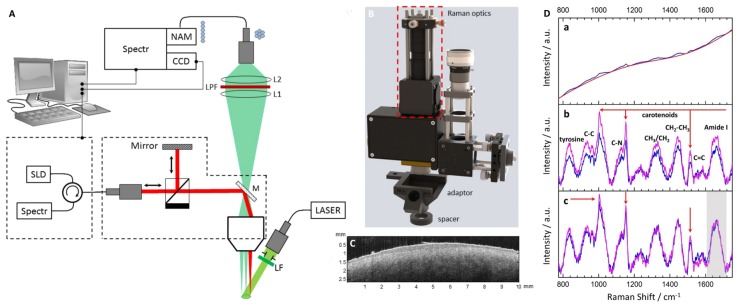
Synopsis of the setup comprising Raman spectroscopy and OCT in the same device. Panel **A** shows a schematic and **B** a photograph of the device. Panels **C** and **D** present an OCT image and Raman spectra respectively which were obtained from in vivo human skin via the combined system. The blue Raman spectrum in panel D was collected from the back of a hand, the purple spectrum originates from the palm. These two spectra are shown as unprocessed data in a, background corrected in b, and furthermore normalized to the Amide I band in c (reproduced with permission from [154]).

In [154], we show that Raman spectroscopy of skin tissue is possible with reasonable signal-to-noise ratio using the collection optics of an OCT system (see Figure 32A,B).

The OCT system is the same as used for the studies presented in the previous Section 4.4.1 and Section 4.4.2. OCT images of tissue (Figure 32C) are recorded as usual but a Raman extension is coupled in straight line to the light path of the collection optics. In this way, detection of Raman scattered photons is realized through the OCT head capitalizing on the fact that the OCT components guide radiation in the spectral range around 1325 nm, the operating spectral range of the OCT, but they are transmissive for visible light. To achieve good collection efficiency the collected Raman scattered light is focused onto a fiber bundle instead of a single fiber for example. The arrangement of the fibers changes from circular at the input end to linear at the output towards the spectrometer. Raman spectra are excited by 532 nm pulsed laser light delivered to the sample by means of an optical fiber. Size and localization of the illuminated spot fills most of the full field of view of the OCT collection optics. We decided for pulsed excitation of Raman spectra as this light source will also serve optoacoustics, the next modality to join the combined device. Visible excitation is expected to provide good nevus contrast for optoacoustic measurement at sufficient penetration depths. Moreover, this standard wavelength is already in the pre-resonance range of most skin carotenoids allowing resonance enhancement of their Raman signal and thus facilitating additional investigations of antioxidant status. Raman spectra detected via the OCT head are presented in Figure 32D. The raw spectra in the upper graph (a) show the strong autofluorescence of tissue in relation to the Raman signals measured with an exposure not exceeding MPE for 532 nm pulsed radiation. Removal of the strong fluorescence background yields the middle spectra (b). The Raman signal from the back of the hand is weaker than that from the palm, presumably due to the stronger (self-)absorption at the back of the hand caused by stronger pigmentation. Normalization to the Amide I bond is applied in (c) assuming similar protein content at both skin sites to set a basis for relative quantitative comparison of the other spectral components. At this normalization, the Raman peaks of the carotenoids are higher at the palm than at the back of the hand indicating higher carotenoid concentration at the palm.

The combination of OCT and Raman spectroscopy provides both morphological and molecular information to support diagnosis in skin cancer screenings and is being complemented by incorporation of optoacoustics allowing determination of invasion depth also for thicker lesions. All three modalities combined have the potential to provide a kind of non-invasive optical biopsy for skin cancer screenings. To succeed in translating optical methods from the physics lab to clinical practice, it is essential to meet future users from medical staff at their level in terms of the information or images they are acquainted with. To this end, we also developed a non-contact dermoscope which not only allows standard application in melanoma screening but is furthermore capable of assessing inflammatory skin diseases [155]. This non-contact dermoscope may be combined with so far non-standard imaging, measurement, and spectroscopic modalities such as OCT, optoacoustics or Raman spectroscopy serving as a visual guide for identification of the ROI (from the dermatologic experience), and for documentation. In this way, we can provide direct connection and comparability of novel and dermatologic standard tools for skin assessment in the future.

## 5. Summary and Outlook

The presented work focuses on the application of optical spectroscopy and imaging to study current research topics in the life sciences spanning from biomedical to environmental issues. Rewarding research objectives have been identified where optical approaches enable non-invasive investigations of molecular contents, molecule kinetics and tissue morphology in vivo and in situ. Multimodal approaches allow mutual benefit as the combined application of optical or hybrid optical and acoustic methods often enable analysis of biological samples extending far beyond the capabilities of the single methods alone. When multimodal approaches are used in imaging, such as the application for a non-invasive biopsy presented here, multimodal measurements are often to be carried out in parallel to enable co-registration of the identical tissue state. However, if dynamics of the biological system are expected to be comparably slow, measurements may also be taken consecutively lowering the technical demands on a combined one-device multimodal system.

Some paths for future research lay out naturally based on the results and achievements reached so far. The combination of OCT, optoacoustics, and Raman spectroscopy for application in dermatology needs to be completed. Together with our non-contact dermoscope, such a multimodal device promises to significantly improve skin cancer screening in two ways: to reduce false positives compared to purely dermoscopic assessment and to reduce the number of re-excisions that are usually necessary because the thickness or invasion depth of the melanoma cannot be determined prior to histopathological assessment by use of dermoscopy alone. The next steps towards this goal mainly involve (pre-)clinical validation of the proposed approach studying a large and representative group with different skin conditions, skin phototype, age, etc.

The successful quantification of optical properties in vivo via OCT and optoacoustics needs to be further developed to improve the estimation of scattering and absorption properties under real tissue conditions. The first step towards this end will be an iterative approach estimating absorption coefficients by optoacoustic measurements neglecting scattering and using them as input parameters for OCT modeling and analysis and vice versa. Based on the developed and available models for light transport in tissue and the comparison of simulated and measured optoacoustic and OCT data, iterative determination of the true, localization dependent optical properties of the probed tissue or other samples may be possible.

Advancing the quantification of molecule concentration via (resonance) Raman spectroscopy will on the one hand be based on improving the scattering model to better describe, for example, scattering on particles and aggregates. Also, the representation of the experimental conditions in the model will be re-evaluated. On the other hand, improved quantification requires reliable input parameters for the tissue optical properties, i.e., parallel work on their faithful non-invasive determination is needed.

Beyond absolute quantification of molecules, work will also continue towards multivariate analysis of Raman spectroscopic data aiming particularly at associating Raman spectra or changes therein to pathophysiological changes in human tissue or body fluids. Here, knowledge about the molecular origin of these changes or absolute quantification of such biomarkers is of course highly interesting, but being able to attempt non-invasive diagnosis even at the basis of Raman spectra of unknown molecular origin would be very helpful. Here, one target application is the correlation of Raman spectroscopic data of human perilymph with pathophysiological conditions of the inner ear. Such application calls for two directions in setting up the analysis. On the one hand, one needs to explore to what extent proteins or groups of proteins may be identified from Raman measurements. If, for example due to increased expression of a biomarker, its Raman fingerprint can be recognized even in the complex composition of the perilymph, this data can support research in causal relationship leading to sensorineural hearing loss. On the other hand, even if decomposition of the perilymph Raman signal is not possible at all, the correlation of variations in the global perilymph fingerprint with pathologic conditions could also contribute significantly to an improved diagnosis of hearing loss. As outlined already, similar correlations are expected with respect to pathologic skin conditions such as melanoma skin cancer.

In general, the ever-increasing computational power is expected to entail great analytic improvements. As seen in this work, numerical modeling is often prerequisite or at least helpful for extracting the desired information from optical data. More computational power enables implementation of increasingly realistic models from light propagation to photochemical dynamics better representing the complexity of research objects in the life sciences. Moreover, machine learning and deep learning concepts may be applied for improved and highly efficient classification of data acquired from multimodal modalities.
